# Viral vector‐based gene therapies in the clinic: An update

**DOI:** 10.1002/btm2.70106

**Published:** 2025-12-29

**Authors:** Kyung Soo Park, Yong In Cho, Samir Mitragotri, Zongmin Zhao

**Affiliations:** ^1^ John A. Paulson School of Engineering and Applied Sciences Harvard University Cambridge Massachusetts USA; ^2^ Wyss Institute for Biologically Inspired Engineering Harvard University Boston Massachusetts USA; ^3^ Department of Pharmaceutical Sciences University of Illinois Chicago Chicago Illinois USA; ^4^ University of Illinois Cancer Center Chicago Illinois USA

**Keywords:** adeno‐associated virus, adenovirus, clinical translation, clinical trials, gene, gene therapy, herpes simplex virus, viral vector

## Abstract

Gene therapy has advanced considerably in recent years, driven by innovations in vector engineering and a more advanced understanding of virology for clinical translation. Since 2021, the U.S. Food and Drug Administration (FDA) has approved seven new viral vector‐based gene therapies, five of which use adeno‐associated virus (AAV) vectors, reinforcing their status as the leading platform for in vivo gene delivery. These approvals encompassed hematologic, neuromuscular, dermatologic, and neurogenetic diseases, using diverse serotypes and delivery routes tailored to the therapeutic context. Disease‐specific patterns of capsid usage reveal advancement in tailored capsid engineering based on anatomical targeting needs. Beyond AAV, non‐AAV vectors, such as herpes simplex virus (HSV) and adenovirus, are actively explored in cancer trials, while lentiviral vectors support applications in oncology and immune‐related disorders. This review provides an updated analysis of the clinical landscape of viral vector‐based gene therapies, highlighting new FDA‐approved products and ongoing clinical trials by vector type, disease indication, and clinical phase since our original review in 2021. Our analysis highlights advances in viral vector technologies that reflect a maturing field, transitioning from proof‐of‐concept studies to precision platforms increasingly capable of addressing rare monogenic disorders and more prevalent, complex diseases.

## Graphical abstract

This review presents an updated overview of the clinical landscape of viral vector‐based gene therapies, highlighting newly approved products and emerging trends in clinical trials.

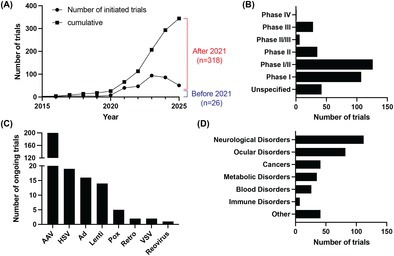

**Translational Impact Statement**

Viral vector‐based gene therapy has progressed from a niche concept to a clinically validated treatment platform, as evidenced by a growing number of FDA approvals and clinical trials. This review provides an up‐to‐date overview of recent developments, including new approvals and trial trends since 2021, highlighting how vector type, delivery method, and disease indication are strategically aligned to optimize therapeutic outcomes. By mapping the evolving clinical landscape, this review identifies emerging trends in vector usage and associated target diseases. The insights presented in this review are intended to support researchers, clinicians, and developers in their translational efforts to advance safe, effective, and durable gene therapies for a broader patient population.

## 1 INTRODUCTION

Current state‐of‐the‐art gene therapies have enabled the achievement of unprecedented efficacies against previously hard‐to‐treat genetic diseases. The delivery of therapeutic vectors has undergone multiple generations of optimizations to lower immune‐related side effects and increase target specificity and the durability of the effects. With the advancements in vector technologies, especially those using viral vectors, safety and efficacy have noticeably improved, driving an avalanche of initiation of clinical trials in recent years, reflected by the growing gene therapy market.

In 2021, we published a review article on the clinical landscape of viral vector‐based gene therapies, which highlighted 13 globally approved products and over 200 clinical trials.^1^ Since then, the field has witnessed several major advancements in new gene therapies for various indications. Over the past 4 years, the U.S. Food and Drug Administration (FDA) has approved 7 new viral gene therapy products for treating various diseases, and over 181 new clinical trials have been initiated. Here, we provide an updated snapshot on the clinical landscape of viral vector‐based gene therapies in 2025, highlight new approvals over the last 4 years, and discuss newly initiated clinical trials. Our analysis focused exclusively on in vivo gene therapy products involving direct viral vector administration and did not include ex vivo engineered cell therapies. By providing an overview of how the field is growing, we aim to suggest the direction in which the field is evolving and advancing.

## 2 NEW GLOBALLY APPROVED PRODUCTS

### 2.1 Landscape and trajectory of global approvals in viral vector gene therapy

The growing portfolio of approved viral vector‐based gene therapies, including those that are approved by the FDA, reflects a significant maturation of the field and its transition into mainstream clinical practice. These approvals span a broad range of disease areas, including hematologic, neuromuscular, retinal, dermatologic, and metabolic disorders, and predominantly target monogenic conditions with well‐characterized genetic etiologies (Table 1). Adeno‐associated virus (AAV) vectors are the most frequently used platform. Products approved to date utilize diverse AAV serotypes with tissue‐specific tropism, demonstrating strategic delivery optimization for various therapeutic targets. Newer AAV serotypes, such as AAVrh74 and its variants, are gaining traction recently, offering enhanced tissue tropism and potentially improved immune evasion. Although AAV dominates the field, the recent approval of a herpes simplex virus (HSV)‐based product for topical gene delivery and an adenovirus‐based product for intravesical instillation signals a diversification in vector platforms and routes of administration.

**TABLE 1:** Updated globally approved viral vector‐based gene therapy products as of 2025.

Product (generic name)	Approval year	Indication	Vector type	Gene delivered	Route of administration	Developer	Administered dose
Products approved on or before 2021							
Gendicine (recombinant human p53 adenovirus)	2003 (China)	Head and neck cancer	Ad5	Wild‐type TP53	Various	Shenzhen SiBiono GeneTech	China (SFDA): typical regimen 1e + 12 viral particles, once weekly × 8 doses (often combined with chemoradiation)
Oncorine (recombinant human adenovirus type 5)	2005 (China)	Nasopharyngeal cancer	Ad5	N/A	Intravenous	Shanghai Sunway Biotech	China (SFDA/NMPA): intratumoral 1–3 vials per day (each 0.5–1.5e + 12 viral particles) for 5 consecutive days; ~3 weeks per cycle
Rexin‐G (retroviral cyclin G1)	2007 (Philippines)	Soft tissue sarcoma, osteosarcoma, pancreatic cancer	Retrovirus	Cytocidal cyclin G1 construct	Intravenous	Epeius Biotechnologies	Philippines: approved locally, but dose not specified in public label; trials used 1–2e + 11 CFU IV 2‐3×/week for 4 weeks per cycle
Glybera (alipogene tiparvovec)	2012 (EU, withdrawn)	Lipoprotein lipase deficiency	AAV1	Human LPL	Intramuscular	uniQure	Single treatment; Max total dose 1e + 12 gc/kg
IMLYGIC (talimogene laherparepvec)	2015 (FDA)	Unresectable melanoma	HSV1	GM‐CSF	Intralesional	Amgen	Initial dose: up to 4 mL at 1e + 6 PFU/mL; second dose: up to 4 mL at 1e + 8 PFU/mL; subsequent doses: Up to 4 mL at 1e + 8 PFU/mL
Luxturna (voretigene neparvovec‐rzyl)	2017 (FDA)	Leber congenital amaurosis	AAV2	Retinoid isomerohydrolase RPE65	Subretinal	Spark Therapeutics	US/EU: 1.5e + 11 vg per eye (0.3 mL subretinal), one eye at a time on separate days (≥6 days apart)
Zolgensma (onasemnogene abeparvovec‐xioi)	2019 (FDA)	Spinal muscular atrophy	AAV9	SMN1 gene	Intravenous	Novartis	US/EU: Single IV infusion of 1.1e + 14 vg/kg
Ervebo (rVSVΔG‐ZEBOV‐GP)	2019 (FDA/EMA)	Ebola virus infection	VSV	Ebola virus GP	Intramuscular	Merck	EU/US: single 1 mL IM dose containing ≥7.2e + 7 PFU of rVSVΔG‐ZEBOV‐GP
Zabdeno/Mvabea (Ad26.ZEBOV/MVA‐BN‐Filo)	2020 (EMA)	Ebola virus infection	Ad26 + MVA	Ebolavirus GP	Intramuscular	Johnson & Johnson	EU: heterologous 2‐dose regimen‐Zabdeno (Ad26.ZEBOV) 0.5 mL IM followed ~8 weeks later by Mvabea (MVA‐BN‐Filo) 0.5 mL IM; viral particle counts not specified in label
AZD1222 (ChAdOx1‐S)	2020 (EMA, others)	COVID‐19	ChAd	SARS‐CoV‐2 Spike(S) protein	Intramuscular	AstraZeneca/Oxford	EU/Global: two 0.5 mL IM doses, each with 2.5e + 8 infectious units of ChAdOx1‐S, given 4–12 weeks apart
JNJ‐78436735 (Ad26.COV2.S)	2021 (FDA/EMA)	COVID‐19	Ad26	SARS‐CoV‐2 Spike (S) protein	Intramuscular	Johnson & Johnson	US/EU (Ad26.COV2.S): single 0.5 mL IM dose containing 5e + 10 viral particles (or not less than 2.5e + 10 vp per WHO SmPC)
Sputnik V (rAd26‐S/rAd5‐S)	2021 (Russia, others)	COVID‐19	Ad26 + Ad5	SARS‐CoV‐2 Spike(S) protein	Intramuscular	Gamaleya Institute	Russia: two 0.5 mL IM doses‐Dose 1 (rAd26‐S) and Dose 2 (rAd5‐S), each ~1e + 11 viral particles, 21 days apart
Convidecia (Ad5‐nCoV)	2021 (China, others)	COVID‐19	Ad5	SARS‐CoV‐2 Spike(S) protein	Intramuscular	CanSino Biologics	China: single 0.5 mL IM dose containing ≥5e + 10 viral particles (Ad5‐nCoV)
Products approved since 2021							
Adstiladrin (nadofaragene firadenovec‐vncg)	2022 (FDA)	Non‐muscle invasive bladder cancer	Ad5	Interferon alfa‐2b	Intravesical instillation	Ferring Pharmaceuticals	US: intravesical 75 mL instillation at 3e + 11 viral particles/mL once every 3 months
Roctavian (valoctocogene roxaparvovec)	2022/2023 (FDA/EMA)	Hemophilia A	AAV5	F8 (Factor VIII)	Intravenous	BioMarin Pharmaceutical	US/EU: single IV infusion of 6e + 13 vg/kg
Hemgenix (etranacogene dezaparvovec)	2022 (FDA)	Hemophilia B	AAV5	Padua variant of FIX (Factor IX)	Intravenous	CSL Behring	US/EU: single IV infusion of 2e + 13 gc/kg
Beqvez (fidanacogene elaparvovec)	2024 (FDA/EMA, others)	Hemophilia B	AAVrh74var	Padua variant of FIX (Factor IX)	Intravenous	Pfizer & Sangamo Therapeutics	US/EU: single IV infusion of 5e + 11 vg/kg
Elevidys (delandistrogene moxeparvovec)	2023 (FDA)	Duchenne muscular dystrophy	AAVrh74	Micro‐dystrophin	Intravenous	Sarepta Therapeutics	US: single IV infusion of 1.33 × 10^14^ vg/kg for <70 kg; fixed 9.31e + 15 vg for ≥70 kg
Vyjuvek (beremagene geperpavec)	2023 (FDA)	Dystrophic epidermolysis bullosa (DEB)	HSV‐1	COL7A1	Topical	Krystal Biotech	US/EU: topical once‐weekly; nominal concentration 5e + 9 PFU/mL; max weekly dose 3.2e + 9 PFU (≥3 years) per FDA materials
Kebilidi (eladocagene exuparvovec)	2024 (FDA/EMA)	AADC deficiency	Modified rAAV2 capsid	DDC	Intracranial	PTC Therapeutics	EU (Upstaza): one‐time bilateral putaminal infusion totaling 1.8e + 11 vg (4 × 0.08 mL; 0.45e + 11 vg per site)

Abbreviations: EMA, European Medicines Agency; GM‐CSF, granulocyte‐macrophage colony‐stimulating factor; LPL, lipoprotein lipase; N/A, not applicable; TP53, tumor protein p53; GP, glycoprotein; RPE, retinal pigment epithelium; SMN, survival motor neuron; SFDA, state food and drug administration; gc, genome count; vg, vector genome; PFU, plaque‐forming units; CFU, colony‐forming units; IV, intravenous; IM, intramuscular.

Recent approvals since 2021 indicate a clear regulatory preference for one‐time, durable treatments that address serious, often rare diseases with limited therapeutic options. These therapies are increasingly utilizing systemic administration to target internal organs, such as the liver or muscle, enabled by capsid engineering and serotype selection. At the same time, localized delivery strategies remain important for certain indications, particularly in ophthalmology and dermatology. The clustering of approvals around hematologic and neuromuscular indications reflects both strong clinical demand and the availability of measurable, gene‐correctable phenotypes. Overall, the trajectory of recent approvals indicates continued momentum toward expanding the scope of gene therapy, both in terms of disease indications and delivery modalities, setting the stage for broader adoption in more complex and prevalent disorders.

### 2.2 Recent advancements in engineered viral vectors

Since 2021, substantial progress in viral vector engineering has advanced gene therapy by leveraging rational design, directed evolution, and machine learning to improve safety, targeting specificity, and delivery efficiency.^2^, ^3^ Capsid engineering remains a central focus, extending beyond natural serotypes through high‐throughput screening and structure‐guided design to generate customized AAV capsids that evade pre‐existing antibodies and achieve tissue‐specific targeting, particularly in the brain.^4^, ^5^, ^6^, ^7^ These efforts aim to enhance local therapeutic efficacy while minimizing systemic exposure and immune activation. Another major direction integrates gene‐editing systems such as CRISPR/Cas9 with AAV and lentiviral platforms to enable precise genomic modification, which is essential for correcting monogenic disorders and mitigating off‐target effects.^8^ Optimization of vector genomes includes refining the genetic payload using self‐complementary AAV (scAAV) constructs for rapid transgene expression, incorporating insulator elements to prevent unintended host‐gene activation, and removing bacterial backbone sequences to improve biosafety.^9^ A recent innovation, acoustically targeted gene delivery, combines engineered viral vectors with focused ultrasound (FUS) to transiently and non‐invasively disrupt biological barriers such as the blood–brain barrier, thereby facilitating precise delivery of therapeutics to the central nervous system.^10^, ^11^

### 2.3 Newly approved products since 2021

Below are brief descriptions of the newly approved viral vector‐based gene therapy products since 2021.

#### 2.3.1 Treatment for bladder cancer

Adstiladrin (nadofaragene firadenovec‐vncg), developed by Ferring Pharmaceuticals, is a non‐replicating adenovirus serotype 5 (Ad5)‐based gene therapy approved by the FDA in 2022 for the treatment of non‐muscle invasive bladder cancer (NMIBC) unresponsive to Bacillus Calmette‐Guérin (BCG). The therapy delivers the human interferon alfa‐2b (IFNα2b) gene directly into the bladder urothelium through intravesical catheter instillation. Local expression of IFNα2b enhances anti‐tumor immune responses and exerts direct antiproliferative effects on tumor cells.^12^, ^13^ Adstiladrin represents a novel therapeutic approach for NMIBC patients who have limited options after BCG failure.

#### 2.3.2 Treatments for hemophilia

Roctavian (valoctocogene roxaparvovec), developed by BioMarin Pharmaceutical, is a gene therapy for hemophilia A, which is characterized by a deficiency in factor VIII clotting activity. It is the first gene therapy approved for this condition in the European Union (with EMA approval in 2022) and recently received FDA approval in 2023. Roctavian uses an AAV5 vector to deliver a functional copy of the F8 gene, encoding factor VIII, a crucial clotting protein in blood.^14^, ^15^, ^16^ The therapy targets the liver, where liver cells produce factor VIII upon administration. The durability of factor VIII expression is being closely monitored, with Phase II and III, 7‐ and 4‐year assessments, respectively, in 2024 reporting sustained benefits over several years, thereby reducing the need for prophylactic factor VIII infusions.^17^, ^18^

Hemgenix (etranacogene dezaparvovec) is a gene therapy for hemophilia B, developed by CSL Behring. It was approved by the FDA in 2022, making it the first gene therapy for hemophilia B characterized by factor IX (FIX) deficiency. It also uses the AAV5 vector to deliver a functional copy of a modified FIX gene called the Padua variant (FIX‐R338L, hFIX Padua), which has 8–10 times higher activity than the wild‐type FIX.^19^ During a Phase III study, patients showed sustained levels of FIX, reaching ~15–60% of the normal value, while the annualized bleeding rates (ABR) were reduced by over 90%.^20^

Beqvez (fidanacogene elaparvovec) is another gene therapy for hemophilia B developed by Pfizer and Sangamo Therapeutics. FDA‐approved in April 2024 based on the Phase III BENEGENE‐2 trial, it is also designed for sustained FIX production to reduce bleeding episodes and the need for regular FIX replacement therapy.^21^ The treatment was generally well‐tolerated in clinical studies without serious adverse reactions. Beqvez uses an AAVrh74 serotype‐derived recombinant viral capsid (AAVRh74var), which can transduce hepatocytes.^22^ Like Hemgenix, it delivers the Padua variant of FIX, with hepatocytes as the primary target. However, in early 2025, Pfizer announced the discontinuation of the global development and commercialization of Beqvez, citing multiple factors, including limited interest from both patients and healthcare providers, as evidenced by the lack of patient uptake since its FDA approval in 2024.^23^ The high price tag for a single dose of Hemgenix and Beqvez, $3.5 million as of 2024, raised concerns among patients and doctors.^24^

#### 2.3.3 Treatment for Duchenne muscular dystrophy

Elevidys (delandistrogene moxeparvovec) is a gene therapy for Duchenne muscular dystrophy (DMD) developed by Sarepta Therapeutics. It was approved by the FDA in 2023 under the Accelerated Approval Program, becoming the first gene therapy for DMD.^25^ DMD is caused by mutations in the DMD gene, which encodes dystrophin, a critical protein for muscle fiber stability related to muscle strength and protection. Elevidys delivers a micro‐dystrophin gene, which was engineered to fit into the AAVrh74 vector and produce a truncated but functional version of dystrophin (called Elevidys micro‐dystrophin).^26^ Since the AAVrh74 capsid has high tropism for striated muscle, including skeletal and cardiac muscles, the treatment is given intravenously.^27^, ^28^, ^29^ Muscle biopsy results showed robust micro‐dystrophin expression, along with improvements in motor function in some patients. Due to three deaths in non‐ambulatory patients, the FDA requested a full pause on Elevidys in July 2025 but later lifted the hold for ambulatory patients. As of August 2025, Sarepta is collaborating with the FDA to update Elevidys' prescribing information to include a black box warning for acute liver injury and acute liver failure.

#### 2.3.4 Treatment for dystrophic epidermolysis bullosa

Vyjuvek (beremagene geperpavec) is an FDA‐approved topical gene therapy developed by Krystal Biotech for treating dystrophic epidermolysis bullosa (DEB), a rare genetic skin disorder. It is the first‐ever topical gene therapy.^30^ DEB is caused by mutations in COL7A1, which encodes type VII collagen (C7), a protein critical for skin integrity. Vyjuvek uses an engineered herpes simplex virus type 1 (HSV‐1) vector that penetrates skin cells at the wound site and restores COL7A1 expression, helping to form anchoring fibrils that improve skin structure.^31^, ^32^ As a topical, non‐integrating gene therapy, Vyjuvek is considered safer than systemic gene therapies due to its limited spread beyond the application site.

#### 2.3.5 Treatment for aromatic L‐amino acid decarboxylase deficiency

Kebilidi (eladocagene exuparvovec‐tneq) is an AAV‐based gene therapy for the treatment of aromatic L‐amino acid decarboxylase (AADC) deficiency in both adult and pediatric patients. This approval, granted in November 2024, marks the first FDA‐approved gene therapy administered directly to the brain. AADC deficiency is a rare genetic disorder caused by mutations in the DDC (dopa decarboxylase) gene, leading to impaired production of the AADC enzyme. This enzyme is crucial for synthesizing neurotransmitters like dopamine and serotonin, essential for communication between nervous systems. Kebilidi functions by delivering a functional copy of the DDC gene directly into the putamen, a brain region involved in motor control.^33^, ^34^ The introduction of the functional gene enables the production of the AADC enzyme, thereby restoring dopamine synthesis and improving motor functions.

### 2.4 Update to the old trials identified as active in 2021

**FIGURE 1:**
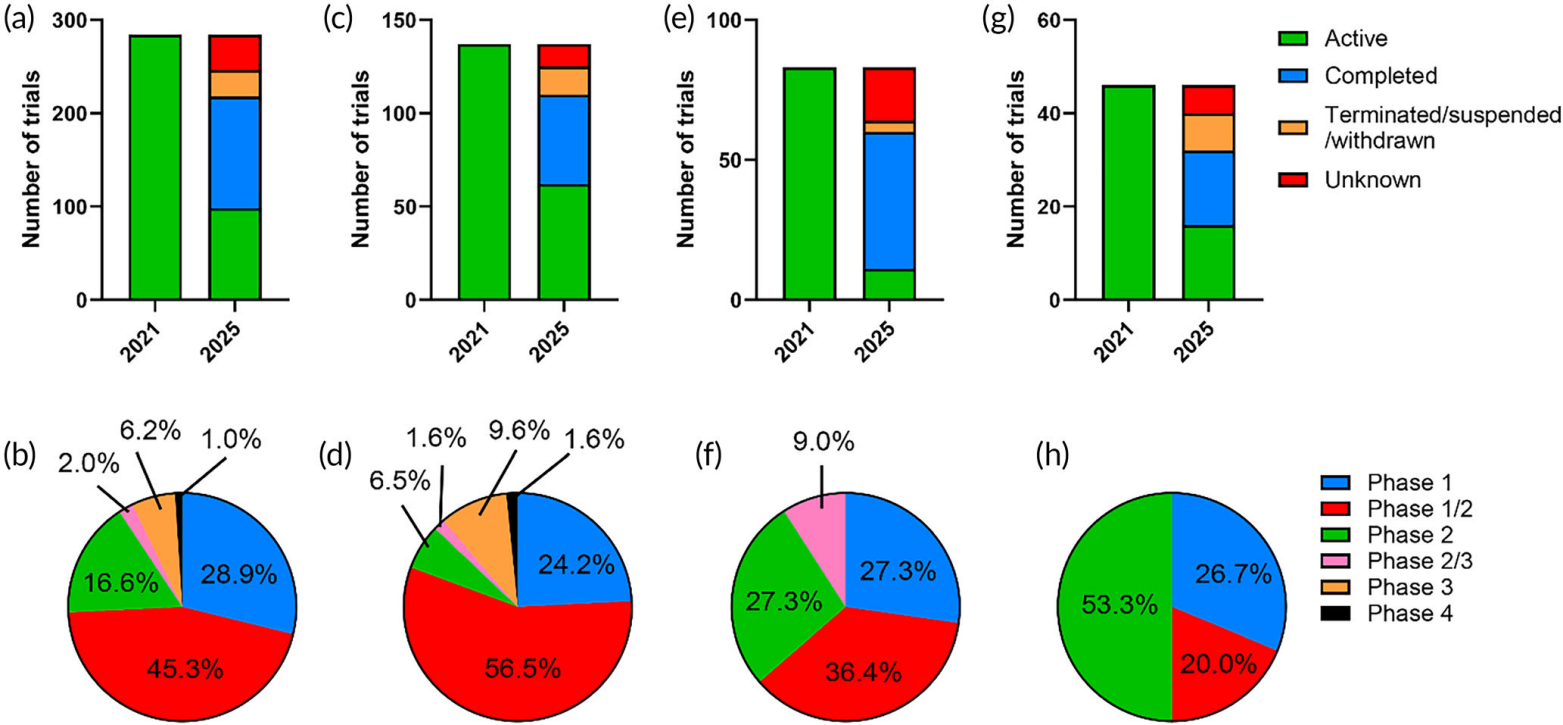
Change of status for the viral vector‐based gene therapy clinical trials identified as active in 2021, which was covered in our previous review article.^1^ The number of (a) total trials, (c) AAV‐based trials, (e) adenovirus‐based trials, and (g) HSV‐based trials with different status in 2021 versus 2025 are shown. The phase distribution of trials remaining active in 2025 from (b) total trials, (d) AAV‐based trials, (f) adenovirus‐based trials, and (h) HSV‐based trials was also analyzed.

In our previous article, we reported 284 viral vector‐based gene therapy trials identified as active in 2021.^1^ Of these, 98 remain ongoing/active, 120 have been completed, and the rest were terminated, suspended, withdrawn, or have unknown status (Figure 1a). Over 90% of the active trials are in Phase I and/or Phase II (Figure 1b). We listed representative examples of terminated trials in Table S1 and provided comments on the primary reasons for termination. We reported a total of 137 active AAV‐based clinical trials as of 2021, spanning a wide range of indications, including ocular, neurological, metabolic, hematological, and musculoskeletal disorders.^1^ Of these, 62 trials remain active, while 48 have been completed, and 15 were terminated, suspended, or withdrawn (Figure 1c). Out of the 62 active trials, 54 are in Phase I and/or Phase II, while 6 are in Phase III (Figure 1d). All terminated trials were in Phase I or Phase II, with the primary reasons being lack of efficacy, safety concerns, or strategic decisions (Table S1). Notably, seven Phase III trials involving five different AAV‐based therapies were listed as completed. These include valoctocogene roxaparvovec for Hemophilia A (NCT03370913, NCT03392974), etranacogene dezaparvovec for Hemophilia B (NCT03569891), onasemnogene abeparvovec‐xioi for spinal muscular atrophy (SMA) (NCT03505099, NCT03837184), lenadogene nolparvovec for Leber Hereditary Optic Neuropathy (NCT03406104), and AAV5‐RPGR for X‐linked Retinitis Pigmentosa (NCT04671433). Several of these trials have reported positive results. For example, the completed Phase III SPR1NT trial (NCT03505099) evaluated onasemnogene abeparvovec, which was FDA‐approved in 2019 for symptomatic SMA, in presymptomatic infants with biallelic *SMN1* mutations treated before 6 weeks of age.^35^ Results showed that early treatment significantly improved motor function, ventilator‐free survival, and independence in feeding and respiration, compared to untreated or late‐treated patients. Early intervention also demonstrated a favorable benefit‐to‐risk profile. The completed Phase III RESTORE trial (NCT03406104) evaluated lenadogene nolparvovec in patients with Leber Hereditary Optic Neuropathy caused by the MT‐ND4 variant.^36^ The therapy showed sustained bilateral improvement in best‐corrected visual acuity (BCVA) and a favorable safety profile for up to 5 years, indicating its long‐term therapeutic potential.

In our 2021 review article, we identified 83 active adenovirus‐based gene therapy clinical trials, primarily focused on cancer treatment and vaccination against infectious diseases.^1^ As of 2025, only 11 of these trials remain active, while 49 have been completed, and 23 have been terminated, suspended, withdrawn, or have unknown status (Figure 1e). Notably, 10 out of the 11 active trials are in Phase I and/or Phase II (Figure 1f). Among the completed trials, 35 targeted vaccinations against SARS‐CoV‐2, Ebola virus, HIV, or tuberculosis, while 13 focused on treating various solid tumors. The completed oncology trials included four Phase II trials, some of which reported positive results. Notably, preliminary positive results were announced for the completed Phase II trial (NCT04416516) evaluating SP‐002, an adenovirus serotype 5 vector encoding human IFN‐γ, in combination with vismodegib (a Hedgehog pathway inhibitor) in patients with multi‐lesional basal cell carcinoma. A single dose of SP‐002 followed by 4 weeks of vismodegib resulted in a 48–75% complete histological clearance (CHC) rate in patients with 1–3 target lesions. The combination therapy also demonstrated a safety profile consistent with those of the individual agents, with no new safety signals observed.

Of the 46 HSV‐based trials identified in our 2021 article,^1^ 16 remain active, 16 have been completed, and 14 were terminated, suspended, withdrawn, or have unknown status (Figure 1g). All of the active trials are in Phase I and/or Phase II (Figure 1h). Notably, 15 of the 16 completed trials focused on talimogene laherparepvec (T‐VEC), an HSV‐1‐based oncolytic virus approved by the FDA in 2015 for unresectable melanoma. These studies aimed to expand its application to other solid tumors, such as breast cancer, pancreatic cancer, and soft tissue sarcoma, or to evaluate its efficacy in combination with other treatments, including chemotherapy, radiotherapy, and immune checkpoint inhibitors. The only completed non‐oncology trial evaluated the topical application of beremagene geperpavec, which was approved by the FDA in 2023 for the treatment of dystrophic epidermolysis bullosa. The majority of terminated HSV trials were also associated with talimogene laherparepvec, with lack of efficacy and challenges in patient recruitment being the primary reasons for termination (Table S1).

## 3 ONGOING CLINICAL TRIALS FOR GENE THERAPIES USING VIRAL VECTORS

Gene therapy, as discussed in this review, refers specifically to in vivo gene delivery methods that directly modify or regulate genetic material within a patient's cells inside the body. This includes approaches such as injection, insertion, or infusion of therapeutic genetic material. By contrast, ex vivo strategies, where cells or tissues are genetically modified outside the body and then reintroduced into the patient, fall outside the scope of this review. Examples of such excluded approaches include stem cell therapies (using modified or unmodified cells for regenerative purposes), T‐cell therapies like CAR‐T (where T cells are engineered externally), and other cellular therapies involving ex vivo genetic manipulation. Comprehensive reviews on these modalities are available elsewhere.^37^ If a therapy edits, replaces, or regulates genes in vivo, it falls under our definition of gene therapy. We searched for ongoing clinical trials on the clinicaltrials.gov website using the keywords “gene therapy” and “gene delivery,” followed by manual screening to exclude irrelevant trials that did not fall under our definition of gene therapy.

### 3.1 Overall landscape of viral vector‐based gene therapy clinical trials

**FIGURE 2:**
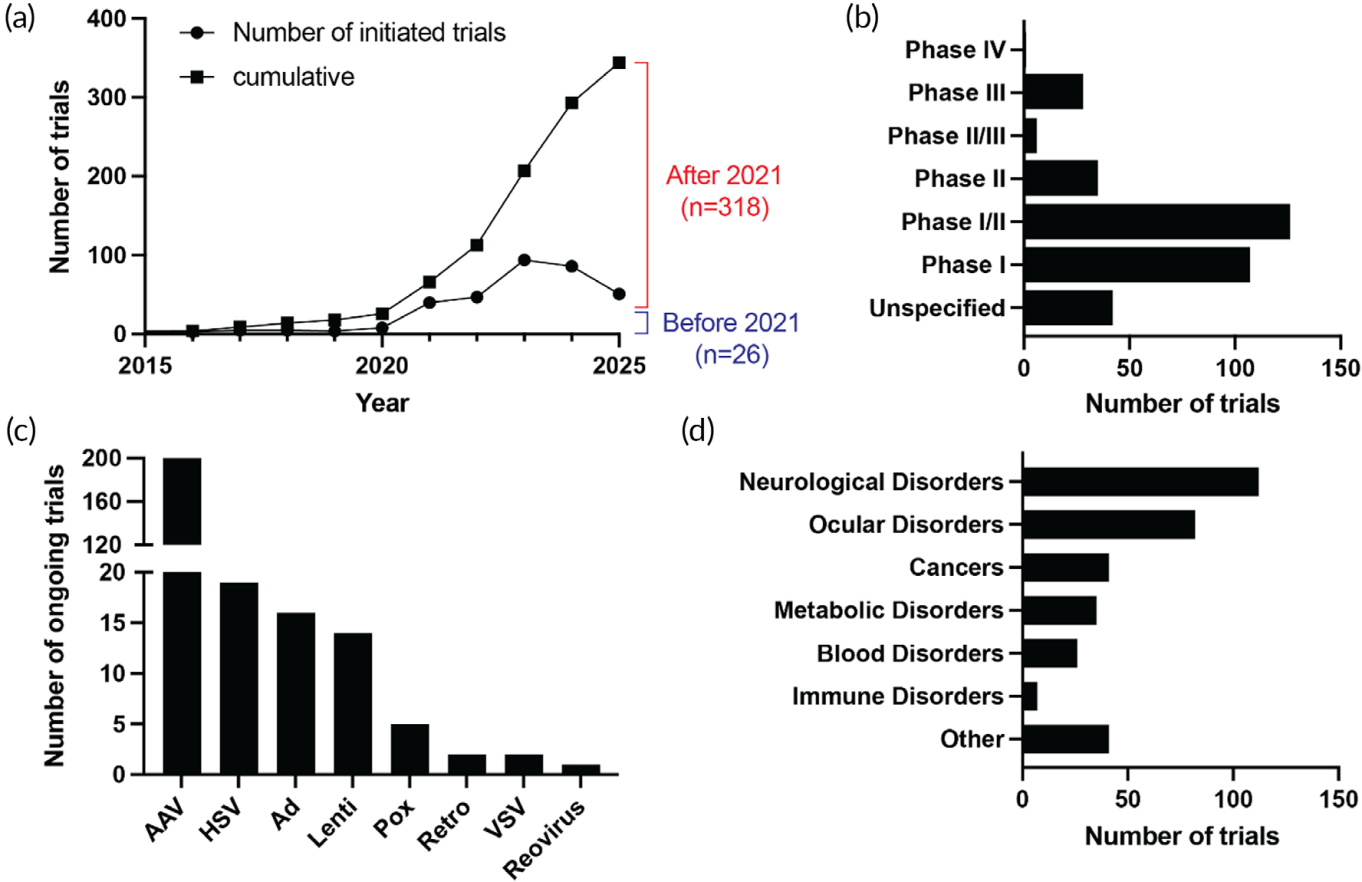
Overall trends in ongoing viral vector‐based gene therapy clinical trials as of 2025. (a) The number of currently ongoing gene therapy trials initiated each year (line dots) and the yearly cumulative numbers (line squares). Cumulative numbers do not account for any terminated trials. Trials initiated before 2021 (*n* = 26) (blue) and after 2021 (*n* = 318) (red). (b) The number of currently ongoing clinical trials by phase, (c) by viral vector type, and (d) by disease category. AAV, adeno‐associated virus; Ad, adenovirus; Pox, poxvirus; Lenti, lentivirus; HSV, herpes simplex virus; VSV, vesicular stomatitis virus; Retro, retrovirus.

The initiation rate of viral vector‐based gene therapy trials has markedly accelerated since 2015, with a sharp surge beginning in 2021 (Figure 2a). Of the 344 trials analyzed, 318 were initiated after 2021, highlighting the recent expansion of the field. This increase likely reflects post‐pandemic growth in biotechnology investment, maturation of viral vector platforms, and greater regulatory clarity for gene therapy development. The number of newly initiated trials peaked in 2023 and remained elevated through 2024, indicating sustained momentum in advancing viral vector‐based therapies.

In terms of clinical development stages, the majority of trials initiated since 2021 are in early phases, particularly Phase I and Phase I/II, underscoring the exploratory nature of most current viral gene therapy programs, given their recent trial start dates (Figure 2b). The relatively smaller number of late‐phase trials indicates that while the field is expanding, many programs remain in pre‐commercial stages.

Next, we analyzed ongoing trials by viral vector type (Figure 2c). Adeno‐associated virus (AAV) continues to dominate the field, owing to its favorable safety profile, broad applicability, and high transduction efficiency in non‐dividing cells. Herpes simplex virus (HSV), adenovirus (Ad), and lentivirus follow as the next most widely used vectors. Poxviral, retroviral, vesicular stomatitis virus (VSV), and reovirus platforms represent smaller shares, each selected for specific properties suited to particular therapeutic applications.

Analysis by therapeutic areas reveals that neurological disorders, ocular disorders, and cancers account for the majority of ongoing efforts (Figure 2d). Metabolic, blood, and immune disorders follow, reflecting the focus on addressing rare monogenic diseases and complex indications through gene therapies.

### 3.2 Disease‐specific trends in viral vector gene therapy trials

**FIGURE 3:**
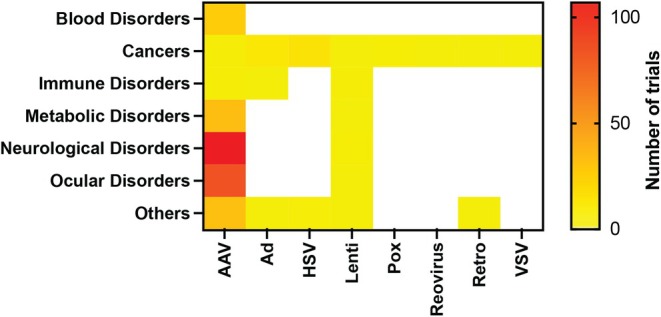
The distribution of delivery modalities across major disease categories. AAV, adeno‐associated virus; Ad, adenovirus; Pox, poxvirus; Lenti, lentivirus; HSV, herpes simplex virus; VSV, vesicular stomatitis virus; Retro, retrovirus.

Disease targeting patterns by vector type reveal platform‐specific preferences across viral vectors (Figure 3). AAVs are primarily used in trials for neurological and ocular disorders, consistent with their clinical success in treating monogenic CNS and retinal diseases. HSV and adenovirus are more frequently used in cancer therapies, owing to their large packaging capacity and ability to trigger robust immune responses. Lentiviral vectors are used across various disease types, including neurological and immune‐related conditions. While AAV remains the dominant platform across most categories, the diversity of vectors in trials implies strategic selection tailored to disease‐specific delivery challenges and therapeutic mechanisms. Below, we describe trends in clinical trials by disease type.

#### 3.2.1 Neurological disorders

Neurological disorders represent the most actively investigated area for viral vector‐based gene therapy clinical trials. Many neurological conditions, such as spinal muscular atrophy (SMA), amyotrophic lateral sclerosis (ALS), and Rett syndrome, are severe, progressive, and lack curative treatments, creating a strong demand for interventions. Many of these disorders are monogenic, making them ideal candidates for viral vector‐based gene replacement strategies. The central nervous system (CNS) is a relatively immune‐privileged environment, which reduces the likelihood of immune‐mediated clearance of the vector.^38^, ^39^ Currently, AAV‐based delivery is dominating this space. In particular, AAV9 is favored due to its brain tropism and ability to induce widespread CNS transduction.^26^, ^27^, ^28^ The clinical success of Zolgensma, an AAV9‐based gene therapy for SMA, has further validated the feasibility and therapeutic potential of CNS‐directed viral gene therapy, catalyzing continued investment and clinical trial expansion in this area.^40^, ^41^

#### 3.2.2 Ocular disorders

Gene therapy for ocular disorders also constitutes a significant proportion of trials. As a small, compartmentalized, and immune‐privileged organ, the eye allows for localized vector administration, often at low doses, with minimal systemic exposure.^42^, ^43^, ^44^ AAV vectors dominate this space, which is consistent with their widespread use in retinal gene therapy. Inherited retinal diseases, such as Leber congenital amaurosis (LCA), retinitis pigmentosa, and X‐linked retinoschisis, are often monogenic and therefore amenable to gene replacement strategies. The landmark approval of Luxturna, the first FDA‐approved AAV‐based gene therapy for a retinal disease, has further accelerated clinical development in ophthalmology.^45^, ^46^, ^47^

#### 3.2.3 Metabolic disorders

Metabolic disorders are emerging as a distinct category of interest in viral vector‐based gene therapy, with a moderate number of clinical trials primarily utilizing AAV vectors. These disorders, which often involve deficiencies in specific enzymes or metabolic pathways, are frequently monogenic and systemic, making them suitable candidates for gene replacement therapies. The liver is typically targeted for transgene expression due to its central role in metabolic regulation and its accessibility to AAV vectors with strong hepatic tropism, such as AAV serotype 8 (AAV8). Conditions like glycogen storage diseases, ornithine transcarbamylase (OTC) deficiency, and phenylketonuria (PKU) are among those being explored in ongoing trials. The ability of AAV‐based vectors to provide durable gene expression after a single administration is particularly advantageous in these chronic diseases, where continuous enzyme replacement is otherwise required.

#### 3.2.4 Blood disorders

In blood disorders, AAV vectors again comprise the majority of delivery platforms used in clinical trials, particularly for hemophilia A and B, where stable hepatic expression of clotting factors can correct systemic deficiency.^48^, ^49^, ^50^ This preference is supported by the liver's natural role in synthesizing clotting factors and the efficient transduction achieved with liver‐tropic serotypes such as AAV8 and AAV5.^51^, ^52^, ^53^ Clinical trials for AAV‐mediated gene therapy in hemophilia have demonstrated sustained transgene expression and significant reductions in annualized bleeding rates, often enabling patients to reduce or eliminate the need for regular prophylactic factor replacement therapy.

While hemophilia remains the primary focus, AAV‐based gene therapies are also expanding into other hematologic indications, including rare coagulation disorders, congenital anemias (e.g., pyruvate kinase deficiency), and complement pathway deficiencies. These applications often rely on liver‐directed expression of therapeutic proteins or regulators, aiming to achieve systemic correction through hepatic gene transfer. The continued success of these programs underscores AAV's clinical utility in hematology and suggests a broader role for liver‐tropic gene delivery platforms in treating systemic, protein‐deficiency‐based disorders.

#### 3.2.5 Cancer

Gene therapy approaches for cancer display the broadest diversity in delivery platforms among all disease categories. Unlike monogenic disorders, cancer gene therapies often aim to stimulate immune responses against tumors or to directly lyse tumor cells. In this context, herpes simplex virus (HSV) is the most frequently used vector, owing to its strong immunogenicity and established use in oncolytic virotherapy. Other vectors, including adenovirus (Ad), poxvirus, reovirus, vesicular stomatitis virus (VSV), lentivirus, and AAV, also appear in trials, reflecting a wide‐ranging exploration of delivery systems to support diverse immunotherapeutic strategies. The inherent immunostimulatory nature of many of these vectors can be advantageous in cancer immunotherapy, where activation of host immune responses is often a key therapeutic mechanism.

### 3.3 Analyses based on vector types

#### 3.3.1 Adeno‐associated virus

Since adeno‐associated virus (AAV) vectors are currently the most investigated vectors tested in clinical trials, we delved deeper into the trends in application. AAV vectors are most commonly used to treat stable monogenic disorders such as hemophilia and retinal dystrophies, owing to their favorable safety profile and ability to drive long‐term gene expression in non‐dividing cells.^54^ In retinal dystrophies, AAVs are particularly effective due to the immune‐privileged nature of the eye, the feasibility of localized delivery via subretinal or intravitreal injection, and the persistent expression achieved in the largely non‐dividing retinal cells.^55^ The clinical success of Luxturna, the first FDA‐approved gene therapy for an inherited retinal disease, has further accelerated the development of AAV‐based therapies in ophthalmology.^47^ Similarly, in hemophilia, AAVs enable stable hepatic expression of clotting factors VIII or IX, leveraging the liver's natural role in producing and providing these proteins into systemic circulation. Liver‐tropic serotypes, such as AAV8 and AAV5, achieve efficient transduction and long‐term expression without genome integration in non‐dividing liver cells.^56^, ^57^, ^58^ Clinical trials have demonstrated durable efficacy with a single intravenous dose, substantially reducing bleeding episodes and the need for ongoing factor replacement therapy.^59^, ^60^

**FIGURE 4:**
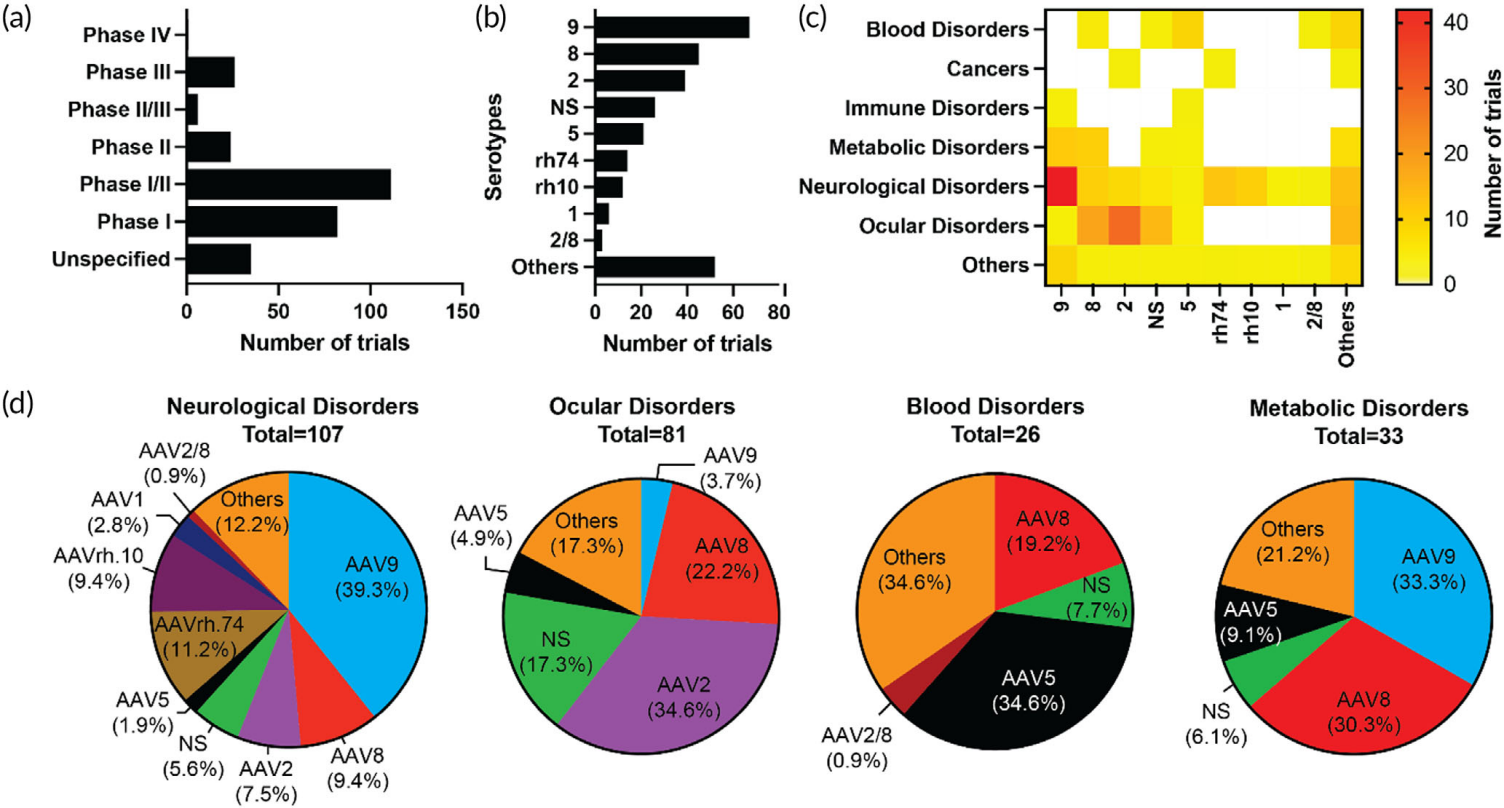
Analysis of AAV serotypes across disease categories in ongoing clinical trials. (a) Distribution of trials by phase. (b) Number of clinical trials by AAV serotype. (c) Distribution of AAV serotypes by disease category. (d) Proportion of serotype usage within each major disease category: neurological disorders, ocular disorders, blood disorders, and metabolic disorders. The total number of trials for each disease category is indicated below each chart. AAV, adeno‐associated virus; NS, not specified.

The list of currently ongoing AAV trials is shown in Table S2. The distribution of AAV‐based clinical trials by phase reveals that most trials are in the early stages of clinical development (Figure 4a). Most are concentrated in Phase I/II, followed by Phase I, indicating a robust pipeline of investigational therapies entering first‐in‐human or dose‐escalation studies. This pattern reflects the ongoing expansion of the AAV field, where many new candidates are still undergoing safety evaluation and exploratory efficacy testing. Expanding on these insights, we analyzed serotype‐specific trends in AAV trials. AAV9 is the most frequently used serotype, followed by AAV8 and AAV2 (Figure 4b). This distribution reflects the intersection of biological performance and regulatory familiarity. AAV9, for instance, is favored for its systemic biodistribution and ability to cross the blood–brain barrier, making it especially suitable for neurological and multisystemic disorders. AAV8, with strong liver tropism, is commonly applied to hemophilia and metabolic disorders, while AAV2 remains widely used in ocular and CNS therapies, often delivered locally.

The heatmap further illustrates how the choice of AAV serotype varies across disease categories (Figure 4c). AAV9 demonstrates widespread usage across most categories, particularly concentrated in neurological disorders, aligning with its CNS‐targeting capabilities. AAV8 also shows substantial usage, particularly in metabolic and blood disorders, likely reflecting its strong liver tropism. Non‐specified (NS) serotypes are frequently observed in neurological, ocular, and blood disorders, suggesting the use of proprietary or engineered capsids with undisclosed identities. Other serotypes, such as AAV2, AAV5, rh74, and rh10, are used more selectively, often in a disease‐ or tissue‐specific context.

Serotype distribution within each disease category highlights these preferences (Figure 4d). In neurological disorders, AAV9 dominates, accounting for nearly 40% of all trials, with additional contributions from AAVrh74 and other serotypes, indicating ongoing optimization for CNS delivery. In ocular disorders, AAV2 capsids are most prevalent, likely due to their historical use in retinal gene therapy and the eye's unique immune privilege.^61^, ^62^ For blood disorders, AAV5 is the most frequently used serotype, followed by AAV8, aligning with liver‐directed gene transfer strategies for systemic protein replacement.^63^, ^64^ In metabolic disorders, AAV8 and AAV9 are the top two serotypes, reinforcing their role in targeting liver and systemic metabolic pathways.

Together, these data suggest that while AAV9, AAV8, and AAV5 remain the clinical workhorses of gene therapy due to their robust efficacy and regulatory track record, there is growing interest in diversifying capsid use. The disease‐specific patterns in serotype usage emphasize how AAV vector selection is dictated by anatomical targeting requirements, vector performance in relevant tissues, and the maturity of supporting preclinical and clinical data. As gene therapy expands into more complex and heterogeneous indications, continued innovation in AAV capsid engineering will be key to overcoming delivery limitations and improving therapeutic precision.

#### 3.3.2 Other viral vectors than AAV

**FIGURE 5:**
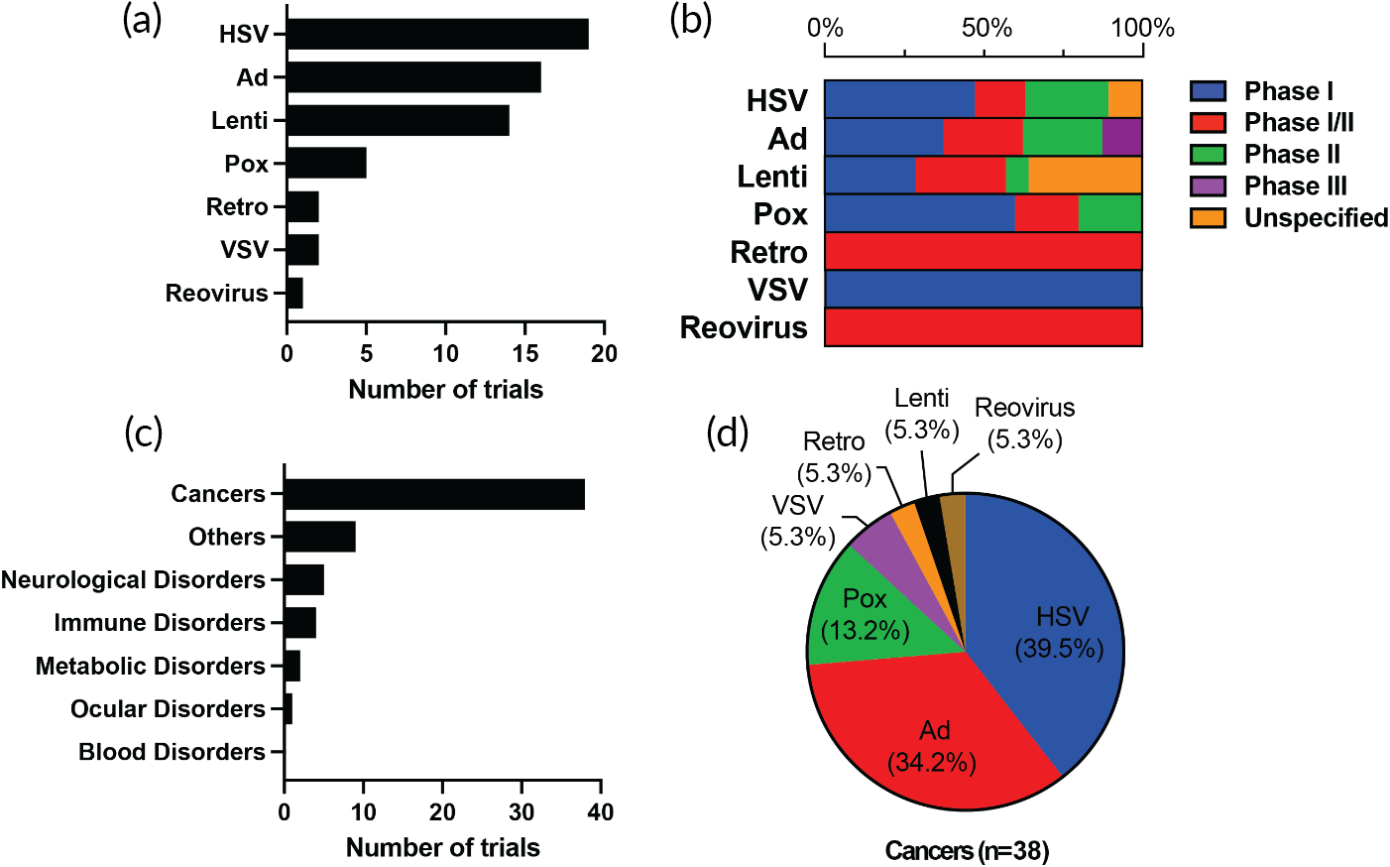
Distribution of clinical trials using non‐AAV viral vectors by vector type, clinical phase, and disease category. (a) Number of trials by viral vector type. (b) Clinical trial phase distribution for each vector. (c) Number of non‐AAV viral vector trials by disease category. (d) Viral vector usage within cancer trials. HSV, herpes simplex virus; Ad, adenovirus; Lenti, lentivirus; Pox, poxvirus; Retro, retrovirus; VSV, vesicular stomatitis virus.

The list of trials that utilize viral vectors other than AAV is shown in Table S3. We investigated the distribution of clinical trials focusing on vector type, clinical development phase, and disease category. Herpes simplex virus (HSV) remains the most commonly used non‐AAV viral vector, followed closely by adenovirus (Ad) and lentivirus, with additional contributions from poxvirus, vesicular stomatitis virus (VSV), retrovirus, and reovirus (Figure 5a). This suggests a continued preference for HSV in applications that benefit from its large packaging capacity and intrinsic immunostimulatory properties, particularly in oncology.^65^ Adenovirus, meanwhile, reflects its long‐standing use in vaccine development and cancer immunotherapy. Next, we sought further insight into the clinical development stages of these vectors (Figure 5b). Most trials involving HSV, Adenovirus, Lentivirus, and poxvirus are concentrated in Phase I or Phase I/II stages, with a small number reaching Phase II or beyond, consistent with their exploratory or immuno‐oncology uses. Lentiviral vectors exhibit a broader phase distribution, including trials with unspecified phases, likely reflecting platform studies in early regulatory review. Retrovirus and reovirus trials remain confined to Phase I/II, indicating low overall trial numbers and limited progression into late‐stage development.

Looking at the number of trials by disease category, most non‐AAV viral vector trials focused on cancer (Figure 5c). Other indications, including neurological, immune, and metabolic disorders, are less frequently represented. Among cancer‐focused trials, HSV accounts for the largest share (39.5%), followed by Ad (34.2%) and poxvirus (13.2%), with lentivirus, retrovirus, VSV, and reovirus each comprising smaller fractions (Figure 5d). This illustrates the diversity of viral platforms under active clinical investigation in oncology. Collectively, these data emphasize the continued versatility and specialization of non‐AAV viral vectors, with HSV and Ad leading in cancer applications, and lentivirus supporting broader use in non‐oncologic indications.

## 4 CONCLUSION

Building upon our earlier efforts to catalog the clinical landscape of viral vector‐based gene therapies in 2021,^1^ this article updates trials since 2021 to capture the most current trends in vector usage, disease targeting, and therapeutic innovation. While AAV vectors remain the predominant modality, particularly in neurological, ocular, and hematologic disorders, our findings reveal a shift toward serotype specialization, such as AAV9 for CNS applications and AAV8 for liver‐directed therapies, reflecting an increasingly nuanced understanding of vector‐tissue interactions. Yet, despite clinical maturity in certain indications, most AAV‐based gene therapies remain in early development stages, underscoring persistent challenges in scalability, immunogenicity, and regulatory clearance. Meanwhile, non‐AAV viral vectors, especially HSV and lentivirus, play expanding roles in oncology and ex vivo gene editing, respectively, highlighting their niche strengths in immunomodulation and stable gene integration.

Looking ahead, AAV vectors are still expected to lead the way in gene delivery, but the next generation will be smarter, safer, and more effective. Capsid engineering through sequence modifications that conceal immune hot spots can reduce antibody or immune cell reactions against AAV.^66^, ^67^ Researchers can now create vast libraries of AAV capsids, test multiple mutations simultaneously, and allow computer algorithms to select the most effective versions. The next generation of capsids is being developed to package efficiently, target human cells more precisely, and evade the immune system in a single design round.^2^, ^6^, ^68^, ^69^ At the same time, single‐cell studies can further guide exactly which capsids bind to which cell types, for example, hepatocytes, immune cells, or blood vessel lining, enabling developers to match each capsid to the disease where it will be most effective.^70^ Additionally, new enhancements to the carrier design can improve safety. Switch‐on promoters keep the therapeutic gene inactive until it reaches the target tissue.^71^, ^72^ Meanwhile, strategic redosing divides the total treatment into smaller, spaced‐out doses or combines with an immunosuppressant to reduce immune responses upon AAV administration.^73^ These approaches, highlighted in a recent review, aim to minimize the high‐dose side effects that have caused some late‐stage trials to fail.^54^

In parallel with viral vectors, an array of non‐viral and hybrid gene delivery systems is advancing rapidly. Liver‐ and lung‐targeted lipid nanoparticles (LNPs) have already delivered base editors and prime‐editing reagents in vivo, including in mice and non‐human primates, achieving therapeutically relevant editing efficiencies with a single systemic dose.^74^, ^75^ Virus‐like particles (VLPs) are emerging as powerful tools for gene editing, offering similar levels of efficiency as AAV and lentivirus, without carrying viral genetic material.^8^ A recent example, the “RIDE” system, allows VLPs to be programmed to target specific cell types such as dendritic cells, T cells, or neurons.^76^ At the same time, extracellular vesicles (EVs), such as exosomes, are being engineered to deliver genetic material like mRNA or siRNA more effectively. For instance, engineered exosomes equipped with peptides have successfully delivered siRNA or CRISPR/Cas9 to neurons in the brain, while others have been modified to reach hard‐to‐access areas of the eye, such as the cornea and retina.^77^, ^78^, ^79^, ^80^ Finally, stimulus‐responsive polymeric nanocarriers and hybrid polymer‐lipid nanoparticles are gaining traction as fully synthetic, re‐dosable alternatives.^81^, ^82^ These systems offer tunable charge density and controlled endosomal escape chemistries (e.g., light‐inducible),^83^ and they can be stealth‐coated (e.g., with PEGylation) to minimize immune recognition.^84^

Collectively, these innovations support the notion that the coming decade will provide a range of fit‐for‐purpose viral, non‐viral, or combinatorial vectors, each customized to a disease's anatomical targets, dosing regimen, and durability needs. This diversity of delivery tools could, in turn, extend gene therapy from specialized cures for ultra‐rare disorders to scalable solutions for more common conditions.

## AUTHOR CONTRIBUTIONS

**Kyung Soo Park**: Conceptualization; data curation; methodology; validation; formal analysis; writing – original draft; writing – review & editing; investigation; visualization. **Yong In Cho**: Formal analysis; data curation; methodology; investigation. **Samir Mitragotri**: Conceptualization; supervision; funding acquisition; project administration; writing – review & editing. **Zongmin Zhao**: Conceptualization; supervision; funding acquisition; project administration; writing – original draft; writing – review & editing.

## CONFLICT OF INTEREST STATEMENT

SM, CP, and ZZ are inventors on patent applications in the field of cell therapies (owned and managed by Harvard University). SM's spouse is an employee and shareholder of Ultrageynix. ZZ is an inventor on patent applications related to gene and cell therapies (owned and managed by University of Illinois Chicago).

## Supporting information


**Table S1.** List of representative trials that were active in 2021 and terminated as of 2025.
**Table S2.** AAV trials.
**Table S3.** Viral vector trials other than AAV.

## Data Availability

All data are available in the main manuscript or Supporting Information.
