# Supplementary material for: Viral vector‐based gene therapies in the clinic: An update
Source: Bioeng Transl Med. 2025 Dec 29;11(1):e70106. doi: 10.1002/btm2.70106 (PMC12821227; doi:10.1002/btm2.70106)
Supplement: Supplementary file 1 — Table S1. List of representative trials that were active in 2021 and terminated as of 2025. Table S2. AAV trials. Table S3. Viral vector trials other than AAV. [file BTM2-11-e70106-s001.docx]

**Supplementary information**

**Table S1.** List of representative trials that were active in 2021 and terminated as of 2025.

**Table S2.** AAV trials.

**Table S3.** Viral vector trials other than AAV.

**Table S1.** List of representative trials that were active in 2021 and terminated as of 2025.

| **Vector type** | **NCT number** | **Name/Sponsor** | **Phase** | **Indication** | **Comment on reason for termination** |
| --- | --- | --- | --- | --- | --- |
| **AAV** | | | | | |
| AAV2/6 | NCT02695160 | SB-FIX/Sangamo Therapeutics | 1 | Hemophilia B | Lack of efficacy |
| AAV2/6 | NCT02702115 | SB-318/Sangamo Therapeutics | 1/2 | MPS I | Lack of efficacy |
| AAV2/6 | NCT03041324 | SB-913/Sangamo Therapeutics | 1/2 | MPS I | Lack of efficacy and severe adverse events |
| AAVS3 | NCT03369444 | FLT180a/Freeline Therapeutics | 1 | Hemophilia B | Administrative reasons |
| AAVS3 | NCT03641703 | FLT180a/Freeline Therapeutics | 1/2 | Hemophilia B | Business/strategic reasons |
| AAV8 | NCT04040049 | FLT-190/Freeline Therapeutics | 1/2 | Fabry Disease, Lysosomal Storage Diseases | Strategic reasons |
| AAV5 | NCT03520712 | Valoctocogene Roxaparvovec/BioMarin Pharmaceutical | 1/2 | Hemophilia A | Lack of efficacy |
| scAAV9 | NCT04088734 | ABO-102/Abeona Therapeutics | 1/2 | MPS IIIA | Lack of efficacy |
| AAV9 | NCT03315182 | rAAV9.CMV.hNAGLU/Abeona Therapeutics | 1/2 | MPS IIIB | Sponsor business/manufacturing decisions |
| AAVHSC15 | NCT03952156 | HMI-102/Homology Medicines | 1/2 | Phenylketonurias, PAH Deficiency | Strategic/financial reasons |
| AAV-LK03 | NCT04581785 | hLB-001/LogicBio Therapeutics | 1/2 | Methylmalonic Acidemia | Insufficient expected benefit |
| **Adenovirus** | | | | | |
| ChAd155 | NCT03866187 | ChAd155-hIi-HBV/GSK | 1/2 | Chronic Hepatitis B | Unknown |
| Ad5 | NCT04406272 | VB11/ Dana-Farber Cancer Institute | 2 | Glioblastoma | Strategic reasons |
| Ad26 | NCT02661464 | Ad26.ZEBOV /Janssen | 3 | Hemorrhagic Fever, Ebola | Logistical/operational reasons |
| **HSV** | | | | | |
| HSV1 | NCT01071941 | rRp450 | 1 | Liver Metastases, Primary Liver Cancers | Unknown |
| HSV1 | NCT04185311 | Talimogene Laherparepvec | 1 | Breast cancer | Logistical participant recruitment issues |
| HSV1 | NCT03300544 | Talimogene Laherparepvec | 1 | Locally Advanced or Metastatic Rectal Cancer | Inadequate patient accrual |
| HSV1 | NCT03597009 | Talimogene Laherparepvec | 1/2 | Malignant Pleural Effusion | Slow patient accrual |
| HSV1 | NCT03921073 | Talimogene Laherparepvec | 2 | Cutaneous Angiosarcoma | Unknown |
| HSV1 | NCT02658812 | Talimogene Laherparepvec | 2 | Recurrent breast cancer | Lack of efficacy |
| HSV1 | NCT02263508 | Talimogene Laherparepvec | 3 | Melanoma | Met pre-specified criteria for futility |

MPS: mucopolysaccharidose; PAH: phenylalanine hydroxylase.

**Table S2.** AAV trials.

| **NCT Number** | **Study Title** | **Phase** | **Disease Category** | **AAV Serotype** |
| --- | --- | --- | --- | --- |
| NCT06024057 | An Expanded Clinical Study Evaluating the AAV2-RPE65 Gene Therapy(LX101) in Patients With LCA |  | Ocular Disorders | 2 |
| NCT04728841 | Gene Therapy for Chinese Hemophilia A |  | Blood Disorders | 8 |
| NCT05040217 | A Clinical Trial of AAV2-BDNF Gene Therapy in Early Alzheimer's Disease and Mild Cognitive Impairment | PHASE1 | Neurological Disorders | 2 |
| NCT04676048 | ASC618 Gene Therapy in Hemophilia A Patients | PHASE1\|PHASE2 | Blood Disorders | 8 |
| NCT03001830 | Gene Therapy for Haemophilia A. | PHASE1\|PHASE2 | Blood Disorders | 2/8 |
| NCT02716246 | Phase I/II/III Gene Transfer Clinical Trial of ScAAV9.U1a.hSGSH | PHASE2\|PHASE3 | Metabolic Disorders | 9 |
| NCT05821959 | Gene Therapy Trial for Otoferlin Gene-mediated Hearing Loss | PHASE1\|PHASE2 | Other | Anc80 (eng) |
| NCT05598333 | Phosphatase Inhibition by Intracoronary Gene Therapy in Subjects with Non-Ischemic NYHA Class III Heart Failure | PHASE2 | Other | 2i8 |
| NCT05984927 | NG101 AAV Gene Therapy in Subjects With Wet Age-Related Macular Degeneration | PHASE1\|PHASE2 | Ocular Disorders | 8 |
| NCT06492876 | Gene Therapy for DME | PHASE1\|PHASE2 | Ocular Disorders | 2 |
| NCT05611424 | Gene Therapy for Wet AMD | PHASE1 | Ocular Disorders | 2 |
| NCT05748873 | Promising ROd-cone DYstrophy Gene TherapY | PHASE1\|PHASE2 | Ocular Disorders | 9 |
| NCT04680065 | GDNF Gene Therapy for Multiple System Atrophy | PHASE1 | Neurological Disorders | 2 |
| NCT06492850 | Gene Therapy for RPGR Gene Mutation-associated X-linked Retinitis Pigmentosa | PHASE1\|PHASE2 | Ocular Disorders | 5 |
| NCT05916391 | Gene Therapy for Diabetic Macular Edema | PHASE1 | Ocular Disorders | 2 |
| NCT05791864 | A First-in-Human, Open-Label, Dose-Escalation Study to Evaluate the Safety and Tolerability of Gene Therapy with TTX-381 for the Ocular Manifestations Associated with Neuronal Ceroid Lipofuscinosis Type 2 (CLN2) Disease | PHASE1\|PHASE2 | Neurological Disorders | 9 |
| NCT04174105 | Gene Transfer Study in Patients With Late Onset Pompe Disease | PHASE1\|PHASE2 | Neurological Disorders | 8 |
| NCT05345171 | Clinical Study of DTX301 AAV- Mediated Gene Transfer for Ornithine Transcarbamylase(OTC) Deficiency | PHASE3 | Metabolic Disorders | 8 |
| NCT06722170 | A Study of EH002 Gene Therapy for Otoferlin Gene Mutation-mediated Hearing Loss |  | Other | 1 |
| NCT06125847 | NGGT006 Gene Therapy for Homozygous Familial Hypercholesterolemia | EARLY_PHASE1 | Metabolic Disorders | 8 |
| NCT06492863 | Gene Therapy(FT-003) for Wet AMD | PHASE1\|PHASE2 | Ocular Disorders | 2 |
| NCT06662188 | JAG201 Gene Therapy Study in Children & Adults with SHANK3 Haploinsufficiency | PHASE1\|PHASE2 | Other | 9 |
| NCT06533579 | Gene Therapy for B-Cell Acute Lymphoblastic Leukemia | PHASE1\|PHASE2 | Cancers | rh74 |
| NCT03466463 | Gene Therapy for Severe Crigler Najjar Syndrome |  | Neurological Disorders | 8 |
| NCT06517888 | Anti-VEGF Gene Therapy Trial for Vestibular Schwannoma | PHASE1\|PHASE2 | Other | Anc80 (eng) |
| NCT05454774 | A Study of FVIII Gene Therapy for Hemophilia A | EARLY_PHASE1 | Blood Disorders | NS |
| NCT06109181 | Gene Therapy for ACM Due to a PKP2 Pathogenic Variant | PHASE1\|PHASE2 | Other | rh10 |
| NCT05878860 | ATSN-201 Gene Therapy in RS1-Associated X-linked Retinoschisis | PHASE1\|PHASE2 | Ocular Disorders | SPR (eng) |
| NCT05224505 | GNT0006 Gene Therapy Trial in Patients With LGMDR9 | PHASE1\|PHASE2 | Other | 9 |
| NCT06641154 | Gene Therapy for Crigler Najjar Syndrome Type I (AlphaCN) | PHASE1\|PHASE2 | Neurological Disorders | 8 |
| NCT05407636 | Pivotal 2 Study of RGX-314 Gene Therapy in Participants With nAMD | PHASE3 | Ocular Disorders | 8 |
| NCT03952637 | A Phase 1/2 Study of Intravenous Gene Transfer With an AAV9 Vector Expressing Human Beta-galactosidase in Type I and Type II GM1 Gangliosidosis | PHASE1\|PHASE2 | Metabolic Disorders | 9 |
| NCT05709288 | Gene Therapy for Hemophilia B Patients Aged 12-18 Years Old | PHASE1 | Blood Disorders | 843 (eng) |
| NCT06199531 | Safety and Efficacy of GS-100 Gene Therapy in Patients With NGLY1 Deficiency | PHASE1\|PHASE2 | Metabolic Disorders | 9 |
| NCT06388200 | A Phase 3 Study Of OCU400 Gene Therapy for the Treatment Of Retinitis Pigmentosa | PHASE3 | Ocular Disorders | 5 |
| NCT06332807 | AAV Gene Therapy Clinical Study in Adult Classic PKU (PHEdom) | PHASE1\|PHASE2 | Metabolic Disorders | 8 |
| NCT04747431 | A Study of PBFT02 in Participants With FTD and Mutations in the Granulin Precursor (GRN) or C9ORF72 Genes | PHASE1\|PHASE2 | Neurological Disorders | 1 |
| NCT05302271 | Phase IA and IB Study of AAVrh.10hFXN Gene Therapy for the Cardiomyopathy of Friedreich's Ataxia | PHASE1 | Neurological Disorders | rh10 |
| NCT04833907 | rAAV-Olig001-ASPA Gene Therapy for Treatment of Children With Typical Canavan Disease | PHASE1\|PHASE2 | Neurological Disorders | Olig001 (eng) |
| NCT05973630 | ATA-200 Dose-escalation Gene Therapy Trial in Patients with LGMDR5 | PHASE1\|PHASE2 | Neurological Disorders | 8 |
| NCT06289452 | Safety and Efficacy Study of IVB102 Injection in Subjects With X-linked Retinoschisis | EARLY_PHASE1 | Ocular Disorders | NS |
| NCT05693142 | AFFINITY DUCHENNE: RGX-202 Gene Therapy in Participants with Duchenne Muscular Dystrophy (DMD) | PHASE2\|PHASE3 | Neurological Disorders | 8 |
| NCT04912843 | Gene Therapy Clinical Trial for the Treatment Of Leber's HereDitary Optic Neuropathy | PHASE2\|PHASE3 | Ocular Disorders | 2 |
| NCT05400330 | Long-Term Follow-up of Gene Therapy for APOE4 Homozygote Alzheimer's Disease | PHASE1 | Neurological Disorders | rh10 |
| NCT06022744 | An Exploratory Clinical Trial Evaluating LX109 Gene Therapy in Patients With nAMD |  | Ocular Disorders | 2 |
| NCT05248230 | 4D-710 in Adult Patients with Cystic Fibrosis | PHASE1\|PHASE2 | Other | A101 (eng) |
| NCT06138639 | A Study of SGT-003 Gene Therapy in Duchenne Muscular Dystrophy (INSPIRE DUCHENNE) | PHASE1\|PHASE2 | Neurological Disorders | SLB101 (eng) |
| NCT04998396 | A Study of AAV9 Gene Therapy in Participants With Canavan Disease (CANaspire Clinical Trial) | PHASE1\|PHASE2 | Neurological Disorders | 9 |
| NCT06198413 | LX102 in Patients With Neovascular Age-Related Macular Degeneration (nAMD) | PHASE1 | Ocular Disorders | 2 |
| NCT06285643 | A Study of AAV2-GDNF in Adults With Moderate Parkinson's Disease (REGENERATE-PD) | PHASE2 | Neurological Disorders | 2 |
| NCT06111638 | Evaluation of the Safety and Efficacy of Hemophilia A Gene Therapy Drugs | PHASE1\|PHASE2 | Blood Disorders | 5 |
| NCT05394064 | A Study to Evaluate Administration of SBT101 Gene Therapy in Adult Patients With Adrenomyeloneuropathy (AMN) | PHASE1\|PHASE2 | Neurological Disorders | 9 |
| NCT06288230 | An Open Label Study of Gene Therapy Product (Vesemnogene Lantuparvovec) in Spinal Muscular Atrophy | PHASE1\|PHASE2 | Other | 9 |
| NCT06597656 | A Gene Transfer Therapy to Evaluate the Safety and Efficacy of Delandistrogene Moxeparvovec (SRP-9001) Following Therapeutic Plasma Exchange (Plasmapheresis) in Participants With Duchenne Muscular Dystrophy (DMD) and Pre-existing Antibodies to AAVrh74 | PHASE1 | Neurological Disorders | rh74 |
| NCT06692712 | Phase 3, Open-label Study to Assess the Efficacy and Safety of a Single Lumbar Intrathecal Administration of MELPIDA in Individuals with Hereditary Spastic Paraplegia Type 50 (SPG50) Versus Matched Prospective Concurrent Controls. | PHASE3 | Neurological Disorders | 9 |
| NCT05822739 | Safety and Efficacy Study of Parkinson's Disease Gene Therapy Drug (BBM-P002) | EARLY_PHASE1 | Neurological Disorders | NS |
| NCT05874310 | Gene Therapy for Subjects With RPGR Mutation-associated X-linked Retinitis Pigmentosa | EARLY_PHASE1 | Ocular Disorders | 5 |
| NCT05858983 | Gene Therapy in Subjects With Biallelic RPE65 Mutation-associated Retinal Dystrophy | PHASE1\|PHASE2 | Neurological Disorders | 2 |
| NCT05930561 | 4D-150 in Patients With Diabetic Macular Edema | PHASE2 | Ocular Disorders | R100 (eng) |
| NCT05230459 | A Study to Evaluate the Safety of AB-1003 (Previously LION-101) in Subjects With Genetic Confirmation of LGMD2I/R9 (Part1) | PHASE1\|PHASE2 | Neurological Disorders | 9 |
| NCT05881408 | A Gene Transfer Therapy Study to Evaluate the Safety and Efficacy of Delandistrogene Moxeparvovec (SRP-9001) in Non-Ambulatory and Ambulatory Participants With Duchenne Muscular Dystrophy (DMD) | PHASE3 | Neurological Disorders | rh74 |
| NCT06292650 | Safety and Efficacy Study of Novel Gene Therapy ZM-02 for Retinitis Pigmentosa Patients | EARLY_PHASE1 | Ocular Disorders | NS |
| NCT06594094 | An Open-label, Multidose Dose-escalation Study to Understand the Safety of CRISPR Gene-editing Therapy and Its Long-Lasting Effects in DMD Patients (MUSCLE) |  | Neurological Disorders | NS |
| NCT05898620 | A Novel, Regulated Gene Therapy (NGN-401) Study for Female Children with Rett Syndrome | PHASE1\|PHASE2 | Neurological Disorders | 9 |
| NCT06063850 | AMT-260 Gene Therapy Study in Adults with Unilateral Refractory Mesial Temporal Lobe Epilepsy | PHASE1\|PHASE2 | Neurological Disorders | 9 |
| NCT06392724 | A Study to Evaluate the Safety and Tolerability of GEN6050X in Duchenne Muscular Dystrophy. | EARLY_PHASE1 | Neurological Disorders | 9 |
| NCT06391736 | Evaluation of the Safety and Efficacy of Late-onset Pompe Disease Gene Therapy Drug | PHASE1\|PHASE2 | Neurological Disorders | 9 |
| NCT06458595 | Safety and Tolerability of KH658 Gene Therapy in Subjects With Neovascular Age-related Macular Degeneration (nAMD) | PHASE1\|PHASE2 | Ocular Disorders | NS |
| NCT05860569 | Safety Evaluation of Gene Therapy Drug in the Treatment of Primary Hypertriglyceridemic Patients With Recurrent Pancreatitis | PHASE1 | Other | 5 |
| NCT04704921 | Pivotal 1 Study of ABBV-RGX-314 (Also Known as RGX-314) Gene Therapy Administered Via Subretinal Delivery One Time in Participants With nAMD | PHASE2\|PHASE3 | Ocular Disorders | 8 |
| NCT05793307 | Evaluation of the Safety and Efficacy of Infantile-onset Pompe Disease Gene Therapy Drug | PHASE1\|PHASE2 | Neurological Disorders | 9 |
| NCT02852213 | A Single-Stage, Adaptive, Open-label, Dose Escalation Safety and Efficacy Study of AADC Deficiency in Pediatric Patients | PHASE1 | Neurological Disorders | 2 |
| NCT05926765 | A Study of AAV2-hAQP1 Gene Therapy in Participants With Radiation-Induced Late Xerostomia | PHASE2 | Other | 2 |
| NCT06066008 | Safety and Efficacy Study of Novel Gene Therapy ZM-01 for X-linked Retinoschisis Patients | EARLY_PHASE1 | Ocular Disorders | NS |
| NCT06152237 | Safety and Efficacy of TSHA-102 in Pediatric Females with Rett Syndrome (REVEAL Pediatric Study) | PHASE1\|PHASE2 | Neurological Disorders | 9 |
| NCT04514653 | RGX-314 Gene Therapy Administered in the Suprachoroidal Space for Participants With Neovascular Age-Related Macular Degeneration (nAMD) (AAVIATE) | PHASE2 | Ocular Disorders | 8 |
| NCT05606614 | Safety and Efficacy of TSHA-102 in Adolescent and Adult Females with Rett Syndrome (REVEAL Adult Study) | PHASE1\|PHASE2 | Neurological Disorders | 9 |
| NCT05657301 | Safety and Tolerability of KH631 Gene Therapy in Participants With Neovascular Age-related Macular Degeneration | PHASE1 | Ocular Disorders | 8 |
| NCT05672121 | Safety and Tolerability of KH631 Gene Therapy in Subjects With Neovascular Age-related Macular Degeneration (nAMD) | PHASE1\|PHASE2 | Ocular Disorders | 8 |
| NCT03326336 | Dose-escalation Study to Evaluate the Safety and Tolerability of GS030 in Subjects With Retinitis Pigmentosa | PHASE1\|PHASE2 | Ocular Disorders | 2 |
| NCT05567627 | Clinical Exploration of Adeno-associated Virus (AAV) Expressing Human Acid Alpha- Glucosidase (GAA) Gene Therapy for Patients With Infantile-onset Pompe Disease |  | Neurological Disorders | 9 |
| NCT06623279 | Open-laBel Dose-escalation Study for CRISPR/cas13- Rna TargetInG THerapy for the Treatment of Neovascular Age-related Macular Degeneration in Phase I Trial | PHASE1 | Ocular Disorders | NS |
| NCT06663878 | An Exploratory Study to Evaluate the Tolerability and Safety of MWAV201 in Subjects With Wilson Disease |  | Metabolic Disorders | 8 |
| NCT06421831 | Evaluation of Safety and Efficacy of Gene Therapy Drug in the Treatment of Spinal Muscular Atrophy (SMA) Type 3 Patients | PHASE1\|PHASE2 | Neurological Disorders | 9 |
| NCT05906953 | Safety and Efficacy Trial of HG004 for Leber Congenital Amaurosis Related to Rpe65 Gene Mutations (STAR) | PHASE1\|PHASE2 | Ocular Disorders | 9 |
| NCT05152732 | Safety and Tolerability of VGB-R04 in Patients With Haemophilia B | EARLY_PHASE1 | Blood Disorders | NS |
| NCT06178432 | Evaluation of the Safety, Tolerability and Efficacy of Gene Therapy Drug for Late Onset Pompe Disease (LOPD) | EARLY_PHASE1 | Neurological Disorders | NS |
| NCT03328130 | Safety and Efficacy Study in Patients With Retinitis Pigmentosa Due to Mutations in PDE6B Gene | PHASE1\|PHASE2 | Ocular Disorders | 2 |
| NCT05805007 | Safety and Tolerability Study of Gene Editing Drug ZVS203e in Participants With Retinitis Pigmentosa | EARLY_PHASE1 | Ocular Disorders | 8 |
| NCT05824169 | Evaluation of Safety and Efficacy of Gene Therapy Drug in the Treatment of Spinal Muscular Atrophy (SMA) Type 1 Patients | PHASE1\|PHASE2 | Neurological Disorders | 9 |
| NCT05901987 | Evaluation of Safety and Efficacy of Gene Therapy Drug in the Treatment of Spinal Muscular Atrophy (SMA) Type 2 Patients | PHASE1\|PHASE2 | Other | 9 |
| NCT06283212 | A Clinical Study to Evaluate the Safety and Efficacy of ETX101, an AAV9-Delivered Gene Therapy in Children With SCN1A-positive Dravet Syndrome | PHASE1\|PHASE2 | Neurological Disorders | 9 |
| NCT06114056 | A Clinical Study Evaluating the Safety, Tolerability, and Initial Efficacy of Single Intravenous Infusion of JWK007 in Patients With Duchenne Muscular Dystrophy (DMD) | PHASE1 | Neurological Disorders | rh74 |
| NCT06761183 | Safety and Preliminary Efficacy of NXL-001 in Patients with Ischemic Stroke | EARLY_PHASE1 | Other | 9 |
| NCT06112275 | A Clinical Study to Evaluate the Safety and Efficacy of ETX101, an AAV9-Delivered Gene Therapy in Children With SCN1A-positive Dravet Syndrome (Australia Only) | PHASE1\|PHASE2 | Neurological Disorders | 9 |
| NCT06061549 | Modulation of SERCA2a of Intra-myocytic Calcium Trafficking in Heart Failure With Preserved Ejection Fraction | PHASE1 | Other | 1 |
| NCT05630651 | The Efficacy and Safety of ZS801 in Chinese Hemophilia B Patients. |  | Blood Disorders | 5 |
| NCT06141460 | Safety and Efficacy of RRG001 Gene Therapy in Subjects With Neovascular Age-related Macular Degeneration (nAMD) | PHASE1\|PHASE2 | Ocular Disorders | NS |
| NCT06291935 | Safety and Tolerability of Intravitreal Administration of VG901 in Patients With Retinitis Pigmentosa Due to Mutations in the CNGA1 Gene | PHASE1 | Ocular Disorders | 2 |
| NCT05197270 | 4D-150 in Patients with Neovascular (Wet) Age-Related Macular Degeneration | PHASE1\|PHASE2 | Ocular Disorders | R100 (eng) |
| NCT05972629 | A Phase 1/Phase 2 Open-label Study to Evaluate the Safety, Tolerability, and Efficacy of a Single Intravenous Administration of SAR444836 in Adult Participants With Phenylketonuria | PHASE1\|PHASE2 | Metabolic Disorders | SNY001 (eng) |
| NCT05641610 | A Study to Evaluate the Safety and Efficacy of ZS801 in Adult Hemophilia B Patients | PHASE1\|PHASE2 | Blood Disorders | 5 |
| NCT06031727 | CRISPR/cas13-medIated RNA TarGeting THerapy for the Treatment of Neovascular Age-related Macular Degeneration Investigator-initiated Trial (SIGHT-I) | EARLY_PHASE1 | Ocular Disorders | NS |
| NCT05616793 | Safety and Tolerability Subretinal OPGx-001 for LCA5-Associated Inherited Retinal Degeneration (LCA5-IRD) | PHASE1\|PHASE2 | Ocular Disorders | 8 |
| NCT06817382 | A Study to Investigate the Safety and Biodistribution of a Single Intrathecal (IT) Injection of INS1201 in Ambulatory Males With Duchenne Muscular Dystrophy (DMD) | PHASE1 | Neurological Disorders | 9 |
| NCT06207552 | Evaluation of the Safety, Tolerability and Efficacy of a Gene Therapy Drug for the Treatment of Pediatric Fabry Disease | EARLY_PHASE1 | Metabolic Disorders | NS |
| NCT06100276 | Safety, Tolerability, and Exploratory Efficacy Study of Intrathecally Administered Gene Therapy AMT-162 in Adult Participants with SOD1 Amyotrophic Lateral Sclerosis (SOD1-ALS) | PHASE1\|PHASE2 | Neurological Disorders | rh10 |
| NCT06345898 | Safety and Efficacy of a Single Subretinal Injection of JWK002 Gene Therapy in Subjects With X-linked Retinoschisis(XLRS) | EARLY_PHASE1 | Ocular Disorders | 8 |
| NCT06646289 | A Follow-on Study for Second-Eye Treatment for Participants Previously Treated With Gene Therapy for X-Linked Retinitis Pigmentosa (XLRP) | PHASE2 | Ocular Disorders | 5 |
| NCT05901480 | An Investigator Initiated Study for OTOV101N+OTOV101C Injection |  | Other | NS |
| NCT04567550 | RGX-314 Gene Therapy Administered in the Suprachoroidal Space for Participants With Diabetic Retinopathy (DR) Without Center Involved-Diabetic Macular Edema (CI-DME) | PHASE2 | Ocular Disorders | 8 |
| NCT05788536 | A Study of DB-OTO, an Adeno-associated Virus (AAV) Based Gene Therapy, in Children/Infants With Hearing Loss Due to Otoferlin Mutations | PHASE1\|PHASE2 | Other | 1 |
| NCT06526923 | A Phase 1/2 Trial of SP-101 for the Treatment of Cystic Fibrosis (CF) | PHASE1\|PHASE2 | Other | 2.5T (eng) |
| NCT06825858 | A Phase I, Open-label, Multicenter, Dose-Escalating Study to Evaluate the Safety and Tolerability of KH658 Gene Therapy in Participants with Neovascular Age-related Macular Degeneration | PHASE1 | Ocular Disorders | NS |
| NCT06699108 | Study to Evaluate the Efficacy and Safety of VGR-R01 Gene Therapy in Patients with Bietti Crystalline Dystrophy | PHASE3 | Neurological Disorders | 8 |
| NCT06191354 | A Clinical Study Evaluating the Safety and Efficacy of SKG0201 Injection in Patients With Spinal Muscular Atrophy Type 1 |  | Neurological Disorders | NS |
| NCT05903794 | A Study of EXG102-031 in Patients With wAMD (Everest) | PHASE1 | Ocular Disorders | NS |
| NCT06246513 | A Trial to Learn More About an Experimental Gene Therapy Called Bidridistrogene Xeboparvovec (SRP-9003) as a Possible Treatment for Limb Girdle Muscular Dystrophy 2E/R4 | PHASE3 | Neurological Disorders | rh74 |
| NCT06228924 | Open-label, Dose Escalation Study of Safety and Preliminary Efficacy of TN-401 in Adults with PKP2 Mutation-associated ARVC | PHASE1 | Other | 9 |
| NCT06460844 | Study to Evaluate Safety of RTx-015 Injection in Retinitis Pigmentosa Patients (ENVISION) | PHASE1 | Ocular Disorders | 7m8 (eng) |
| NCT06818838 | A Clinical Study Evaluating LY-M001 Injection in the Treatment of Adult Patients with Type I Gaucher Disease | PHASE1\|PHASE2 | Metabolic Disorders | 8 |
| NCT06237777 | A Clinical Study Evaluating the Safety, Tolerability and Initial Efficacy of SKG0106 Intravitreal Injection in Diabetic Macular Edema (DME) Patients | PHASE1 | Ocular Disorders | NS |
| NCT06217861 | A Study to Evaluate the Tolerability, Safety and Efficacy of VGM-R02b | PHASE1 | Metabolic Disorders | 9 |
| NCT06739434 | GCB-002 in Treatment of Patients with Rett Syndrome |  | Neurological Disorders | 9 |
| NCT06224660 | Modulation of SERCA2a of Intra-Myocytic Calcium Trafficking in Cardiomyopathy Secondary to Duchenne Muscular Dystrophy | PHASE1 | Neurological Disorders | 1 |
| NCT06519552 | A Clinical Study Evaluating the Safety, Tolerability, and Initial Efficacy of JWK008 in Patients With Mucopolysaccharidosis Type I | PHASE1 | Metabolic Disorders | 5 |
| NCT06641895 | Evaluation of the Safety and Efficacy of BBM-D101 to Treat Patients With Duchenne Muscular Dystrophy | EARLY_PHASE1 | Neurological Disorders | NS |
| NCT06293729 | Safety and Efficacy Study of NGGT006 in Refractory Hypercholesterolemia Patients | EARLY_PHASE1 | Metabolic Disorders | NS |
| NCT06650319 | A Clinical Study to Evaluate the Safety and Efficacy of LY-M003 Injection in Patients with Wilson Disease | EARLY_PHASE1 | Metabolic Disorders | 8 |
| NCT06272149 | An Exploratory Clinical Trial of VGN-R08b in Patients With Type II Gaucher Disease | EARLY_PHASE1 | Metabolic Disorders | 9 |
| NCT06064890 | A Study to Evaluate the Safety and Effect of AVB-101, a Gene Therapy Product, in Subjects With a Genetic Sub-type of Frontotemporal Dementia (FTD-GRN) | PHASE1\|PHASE2 | Neurological Disorders | 9 |
| NCT06370351 | A Phase I/II Clinical Trial with SENS-501 in Children Suffering from Severe to Profound Hearing Loss Due to Otoferlin (OTOF) Mutations | PHASE1\|PHASE2 | Other | 2 |
| NCT04875754 | A Study Evaluating the Safety, Tolerability, and Range of Biologically Active Doses of ICM-203 in Mild to Moderate Knee Osteoarthritis | PHASE1\|PHASE2 | Other | 2.5 (eng; 1/2) |
| NCT05518188 | Melpida: Recombinant Adeno-associated Virus (serotype 9) Encoding a Codon Optimized Human AP4M1 Transgene (hAP4M1opt) | PHASE1\|PHASE2 | Neurological Disorders | 9 |
| NCT06749639 | Safety and Efficacy Study of PUMCH-E101 Injection in Subjects with RDH12 Retinopathy | EARLY_PHASE1 | Ocular Disorders | NS |
| NCT06003387 | Efficacy and Safety of CSL222 (Etranacogene Dezaparvovec) Gene Therapy in Adults With Hemophilia B With Pretreatment Adeno-associated Virus Serotype 5 (AAV5) Neutralizing Antibodies (Nabs) | PHASE3 | Blood Disorders | 5 |
| NCT06412224 | The Preliminary Safety and Efficacy of RRG001 After Vitrectomy in Subjects With Proliferative Diabetic Retinopathy (PDR) | EARLY_PHASE1 | Ocular Disorders | NS |
| NCT06128564 | A Gene Delivery Study to Evaluate the Safety and Expression of Delandistrogene Moxeparvovec in Participants Under the Age of Four With Duchenne Muscular Dystrophy (DMD) | PHASE2 | Neurological Disorders | rh74 |
| NCT06255782 | An Open-label Study to Investigate ECUR-506 in Male Babies Less Than 9 Months of Age With Neonatal Onset OTC Deficiency (OTC-HOPE) | PHASE1\|PHASE2 | Metabolic Disorders | rh79 |
| NCT04884594 | AAV8-hCocH for Cocaine Use Disorder | PHASE1 | Other | 8 |
| NCT06615206 | A First-in-Human Clinical Trial to Evaluate the Safety, Tolerability, and Efficacy of a Novel CRISPR RNA-editing Therapy in Patients with Mecp2 Duplication Syndrome, a Rare Orphan Disease (HERO) |  | Neurological Disorders | NS |
| NCT05986864 | Phase I/II Study of SKG0106 Intravitreal Injection in Patients With Neovascular Age-related Macular Degeneration (nAMD) | PHASE1\|PHASE2 | Ocular Disorders | NS |
| NCT06518005 | Efficacy and Safety of GNT0003 Following Imlifidase Pre-treatment in Severe Crigler-Najjar Syndrome | PHASE2 | Neurological Disorders | 8 |
| NCT06591793 | Study of Subretinally Injected AAVB-081 in Patients With Usher Syndrome Type IB (USH1B) Retinitis Pigmentosa | PHASE1\|PHASE2 | Ocular Disorders | 8 |
| NCT06775483 | Evaluation of JSKN016 in the Treatment of Advanced Non-small Cell Lung CanceÔºö a Phase II Clinical Study | PHASE2 | Cancers | Not Specified |
| NCT04120493 | Safety and Proof-of-Concept (POC) Study with AMT-130 in Adults with Early Manifest Huntington's Disease | PHASE1\|PHASE2 | Neurological Disorders | 5 |
| NCT04850118 | A Clinical Trial Evaluating the Safety and Efficacy of a Single Subretinal Injection of AGTC-501 in Participants With XLRP | PHASE2\|PHASE3 | Ocular Disorders | 2 |
| NCT06120582 | Study of the Safety, Pharmacodynamics and Efficacy of ANB-002 in Patients With Hemophilia B (SAFRAN) | PHASE1\|PHASE2 | Blood Disorders | 5 |
| NCT05836259 | Multi-center, Open-label, Single-ascending Dose Study of Safety and Tolerability of TN-201 in Adults With Symptomatic MYBPC3 Mutation-associated HCM | PHASE1\|PHASE2 | Other | 9 |
| NCT06061614 | Safety and Efficacy Study of NGGT002 in PKU Adult Subjects | EARLY_PHASE1 | Metabolic Disorders | 8 |
| NCT06743646 | Efficacy and Safety of ZVS101e in Patients With Bietti 's Crystalline Dystrophy | PHASE3 | Other | 2/8 |
| NCT05454566 | A Study Evaluating the Safety, Tolerability, and Activity of ICM-203 in Subjects With Knee Osteoarthritis. | PHASE1\|PHASE2 | Other | 5 |
| NCT06238908 | Safety and Efficacy Study of NGGT003 in Hemophilia A Patients | EARLY_PHASE1 | Blood Disorders | 8 |
| NCT06700096 | An Open-Label, Comparative Study of the Efficacy, Safety and Pharmacodynamics of Single Dose of ANB-002 in Patients with Hemophilia B | PHASE3 | Blood Disorders | 5 |
| NCT06432140 | A Trial to Evaluate Safety and Efficacy of a Product Named VGN-R09b in Severe AADC Deficiency | PHASE1 | Neurological Disorders | 9 |
| NCT05714904 | Safety and Tolerability of ZVS101e in Patients With Bietti 's Crystalline Dystrophy | EARLY_PHASE1 | Neurological Disorders | 2/8 |
| NCT06270316 | Safety, PK/PD, and Exploratory Efficacy Study of AMT-191 in Classic Fabry Disease | PHASE1\|PHASE2 | Metabolic Disorders | 5 |
| NCT06467344 | Study to Evaluate ACDN-01 in ABCA4-related Retinopathy (STELLAR) | PHASE1\|PHASE2 | Ocular Disorders | NS |
| NCT05335876 | Long-term Follow-up of Patients With Spinal Muscular Atrophy Treated With OAV101 in Clinical Trials | PHASE3 | Other | 9 |
| NCT05694598 | Safety and Tolerability of VGR-R01 for Patients With Bietti Crystalline Dystrophy | PHASE1 | Neurological Disorders | 8 |
| NCT06185335 | A Trial of the Safety and Efficacy of Single-Dose Administration of ANB-010 in Subjects With Hemophilia A | PHASE1\|PHASE2 | Blood Disorders | 6 |
| NCT06645197 | An IIT Clinical Study to Evaluate the Safety, Tolerability, and Preliminary Efficacy of a Single Intrathecal Injection of SNUG01 in Patients with Amyotrophic Lateral Sclerosis | EARLY_PHASE1 | Neurological Disorders | 9 |
| NCT05885412 | A Phase 1, Dose Escalation Trial of RP-A601 in Subjects With PKP2 Variant-Mediated Arrhythmogenic Cardiomyopathy (PKP2-ACM) | PHASE1 | Other | rh74 |
| NCT05765981 | An Early Clinical Trial to Evaluate VGN-R09b for Treatment of Aromatic L-amino Acid Decarboxylase (AADC) Deficiency. | EARLY_PHASE1 | Neurological Disorders | 9 |
| NCT04127578 | Phase 1/2a Clinical Trial of PR001 (LY3884961) in Patients With Parkinson's Disease With at Least One GBA1 Mutation (PROPEL) | PHASE1\|PHASE2 | Neurological Disorders | 9 |
| NCT04408625 | Phase 1/2 Clinical Trial of LY3884963 in Patients With Frontotemporal Dementia With Progranulin Mutations (FTD-GRN) | PHASE1\|PHASE2 | Neurological Disorders | 9 |
| NCT06185673 | A Study to Evaluate the Safety and Clinical Activity of Intramuscular Doses of BB-301 Administered to Subjects With Oculopharyngeal Muscular Dystrophy With Dysphagia | PHASE1\|PHASE2 | Neurological Disorders | 9 |
| NCT04179643 | AB-1002 in Patients With Class III Heart Failure | PHASE1 | Other | NS |
| NCT07011771 | A Clinical Trial of CAP-003 Gene Therapy in Adult Patients With GBA1 Associated Parkinson's Disease | PHASE1\|PHASE2 | Neurological Disorders | eng |
| NCT06983158 | A Clinical Trial of CAP-002 Gene Therapy in Pediatric Patients With Syntaxin-Binding Protein 1 (STXBP1) Encephalopathy | PHASE1\|PHASE2 | Neurological Disorders | eng |
| NCT05152823 | Gene Therapy for IGHMBP2-Related Diseases | PHASE1\|PHASE2 | Neurological Disorders | 9 |
| NCT05417126 | Safety and Effects of a Single Intravitreal Injection of vMCO-010 Optogenetic Therapy in Subjects With Stargardt Disease | PHASE2 | Ocular Disorders | 2 |
| NCT05541627 | A Study to Evaluate AB-1001 Striatal Administration in Adults With Early Manifest Huntington's Disease | PHASE1\|PHASE2 | Neurological Disorders | rh10 |
| NCT05740761 | Gene Editing as a Therapeutic Approach for Rett Syndrome |  | Neurological Disorders |  |
| NCT06048185 | Non-interventional Long Term Follow-up Study of Participants Previously Enrolled in the STARLIGHT Study |  | Ocular Disorders | 2 |
| NCT06162585 | Non-Interventional Long Term Follow-up Study of Participants Previously Enrolled in the RESTORE Study |  | Ocular Disorders | 2 |
| NCT04945772 | Efficacy and Safety of MCO-010 Optogenetic Therapy in Adults With Retinitis Pigmentosa [RESTORE] | PHASE2 | Ocular Disorders | 2 |
| NCT06996756 | Gene Therapy for Alpha-1 Antitrypsin Deficiency | PHASE1 | Metabolic Disorders | 8 |
| NCT05121376 | A Gene Therapy Study of BMN 331 in Subjects With Hereditary Angioedema | PHASE1\|PHASE2 | Immune Disorders | 5 |
| NCT04693598 | Gene Transfer Clinical Trial for Krabbe Disease | PHASE1\|PHASE2 | Neurological Disorders | rh10 |
| NCT04817462 | Liver Biopsy In Haemophilia Gene Therapy |  | Blood Disorders |  |
| NCT05932914 | Liver Biopsy Following Gene Therapy For Hemophilia |  | Blood Disorders |  |
| NCT05445323 | Gene Therapy for Cardiomyopathy Associated With Friedreich's Ataxia | PHASE1\|PHASE2 | Neurological Disorders | rh10 |
| NCT05523128 | The Efficacy and Safety of ZS802 in Chinese Hemophilia A Patients. |  | Blood Disorders |  |
| NCT05441553 | A Study to Evaluate the Safety and Efficacy of VGB-R04 in Adult Hemophilia B Patients | PHASE1\|PHASE2 | Blood Disorders | 8 |
| NCT05894343 | Long-term Follow-up of Glutamic Acid Decarboxylase (GAD) Gene Transfer in Parkinson's Disease | PHASE1\|PHASE2 | Neurological Disorders | 2 |
| NCT05228145 | Gene Therapy Study for Children With CLN5 Batten Disease | PHASE1\|PHASE2 | Neurological Disorders | 9 |
| NCT07048808 | An Investigational Trial to Evaluate the Effects of the Drug XC001 Delivered Via an Endocardial Delivery Catheter in Subjects With Chronic Angina Caused by Coronary Artery Disease | PHASE2 | Others | 8 |
| NCT06696456 | Long Term Follow-up Study of AAVAnc80-hOTOF Gene Therapy |  | Neurological Disorders | Anc80 (eng) |
| NCT06297486 | Study of a Gene Therapy Treatment for Hemophilia A | PHASE3 | Blood Disorders | LK03 (eng) |
| NCT06826612 | A Randomized Study of SPK-10001 Gene Therapy in Participants With Huntington's Disease | PHASE1\|PHASE2 | Neurological Disorders |  |
| NCT05603312 | A Double-blind Study to Evaluate the Safety of Glutamic Acid Decarboxylase Gene Transfer in Parkinson's Participants | PHASE1\|PHASE2 | Neurological Disorders | 2 |
| NCT06980948 | Safety and Tolerability Study of ST-503 for Small Fiber Neuropathy-associated Refractory Pain | PHASE1 | Neurological Disorders | 9 |
| NCT06270719 | An Observational Study Comparing Delandistrogene Moxeparvovec With Standard of Care in Participants With Duchenne Muscular Dystrophy |  | Neurological Disorders | rh74 |
| NCT07063030 | A Study of LX107 Gene Therapy in AIPL1-IRD Patients | EARLY_PHASE1 | Ocular Disorders |  |
| NCT06224907 | Phase 3 Study for Efficacy and Safety Outcomes Data in Japanese Patients With Severe Hemophilia A | PHASE3 | Blood Disorders | 5 |
| NCT06088992 | Leber Congenital Amaurosis Inherited Blindness of Gene Therapy Trial(LIGHT) | EARLY_PHASE1 | Ocular Disorders | 9 |
| NCT04783181 | A Study of Gene Therapy for Classic Congenital Adrenal Hyperplasia (CAH) | PHASE1\|PHASE2 | Metabolic Disorders | 5 |
| NCT06544798 | Long-term Follow-up of Gene Therapy for Radiation-Induced Xerostomia | PHASE2 | Cancers | 2 |
| NCT04832724 | RGX-314 Gene Therapy Pharmacodynamic Study for Neovascular Age-related Macular Degeneration (nAMD) | PHASE2 | Ocular Disorders | 8 |
| NCT06948019 | Safety and Efficacy of AAV9/AP4B1 (BFB-101) For Patients With AP4B1-related Hereditary Spastic Paraplegia Type 47 (SPG47) | PHASE1\|PHASE2 | Neurological Disorders | 9 |
| NCT07053358 | Safety and Efficacy Evaluation of LX111 Gene Therapy in nAMD Patients | EARLY_PHASE1 | Ocular Disorders |  |
| NCT05506254 | Long-term Follow-up Study of Patients Who Received hLB-001 Gene Therapy |  | Metabolic Disorders | LK03 (eng) |
| NCT06921317 | GVB-2001 Gene Therapy Via Intracameral Injection for the Treatment of Primary Open Angle Glaucoma | PHASE1\|PHASE2 | Ocular Disorders | 2 |
| NCT05689164 | A Study to Understand the Long-term Safety and Effects of an Experimental Gene Therapy for Duchenne Muscular Dystrophy. | PHASE3 | Neurological Disorders | 9 |
| NCT04884815 | A Phase 1/2/3 Study of UX701 Gene Therapy in Adults With Wilson Disease | PHASE1\|PHASE2 | Metabolic Disorders | 9 |
| NCT04581785 | Gene Therapy With hLB-001 in Pediatric Patients With Severe Methylmalonic Acidemia | PHASE1\|PHASE2 | Metabolic Disorders | LK03 (eng) |
| NCT07058662 | A Clinical Study to Evaluate the Safety, Tolerability, and Efficacy of BBM-D101 in the Treatment of Duchenne Muscular Dystrophy. | PHASE1\|PHASE2 | Neurological Disorders |  |
| NCT05324943 | A Gene Therapy Study in Patients With Gaucher Disease Type 1 | PHASE1 | Metabolic Disorders | S3 (eng) |
| NCT05203679 | Evaluation of the Safety and Efficacy of Hemophilia B Gene Therapy Drug | PHASE2\|PHASE3 | Blood Disorders | 1/6 (eng) |
| NCT05739643 | Gene Transfer Clinical Trial for Infantile and Late Infantile Krabbe Disease Treated Previously With HSCT | PHASE1\|PHASE2 | Neurological Disorders | rh10 |
| NCT06092034 | A Gene Therapy Study of RP-A501 in Male Patients With Danon Disease | PHASE2 | Metabolic Disorders | 9 |
| NCT05096221 | A Gene Transfer Therapy Study to Evaluate the Safety and Efficacy of Delandistrogene Moxeparvovec (SRP-9001) in Participants With Duchenne Muscular Dystrophy (DMD) | PHASE3 | Neurological Disorders | rh74 |
| NCT06300476 | Safety and Efficacy of a Single Subretinal Injection of JWK006 Gene Therapy in Subjects With Stargardt Disease(STGD1) | PHASE1\|PHASE2 | Ocular Disorders | 8 |
| NCT05399069 | Safety and Tolerability of VGR-R01 in Patients With Bietti Crystalline Dystrophy | EARLY_PHASE1 | Ocular Disorders | 8 |
| NCT06614569 | Long-Term Follow-Up of Subjects Treated With AXO-AAV-GM2 for Tay-Sachs or Sandhoff Disease |  | Neurological Disorders | rh8 |
| NCT06275620 | A Study Comparing Two Doses of AGTC-501 in Male Participants With X-linked Retinitis Pigmentosa Caused by RPGR Mutations (DAWN) | PHASE2 | Ocular Disorders | 2 |
| NCT05139316 | A Study of Adeno-Associated Virus Serotype 8-Mediated Gene Transfer of Glucose-6-Phosphatase in Patients With Glycogen Storage Disease Type Ia (GSDIa) | PHASE3 | Metabolic Disorders | 8 |
| NCT05442528 | Lead-in Study of VGB-R04 Gene Therapy for Hemophilia B-- An Observational Survey Analysis Study |  | Blood Disorders | 8 |
| NCT03520751 | Phase I/IIa Trial of scAAV1.tMCK.NTF3 for Treatment of CMT1A | PHASE1\|PHASE2 | Neurological Disorders | 1 |
| NCT04737460 | Study for the Treatment for CLN7 Disease | PHASE1 | Neurological Disorders | 9 |
| NCT06922890 | First-in-Human Clinical Trial of STUP-001, an In Vivo Direct Cell Conversion Gene Therapy for AIS-A/B Chronic Spinal Cord Injury | PHASE1\|PHASE2 | Neurological Disorders | rh10 |
| NCT04798235 | First-in-Human Study of TSHA-101 Gene Therapy for Treatment of Infantile Onset GM2 Gangliosidosis | PHASE1\|PHASE2 | Neurological Disorders | 9 |
| NCT07014020 | RB001 Gene Therapy Study in Children With SHANK3-related Phelan McDermid Syndrome (PMS) |  | Neurological Disorders |  |
| NCT06008938 | An Observational Cohort Study to Characterize the Effectiveness and Safety of HEMGENIX® in Patients With Hemophilia B |  | Blood Disorders | 5 |
| NCT06952686 | A Study of SRP-9005 in Limb Girdle Muscular Dystrophy Type 2C/R5 Pediatric and Adult Participants | PHASE3 | Neurological Disorders | rh74 |
| NCT06213038 | A Clinical Study Evaluating the Safety and Efficacy of SKG0106 in Patients With Neovascular Age-related Macular Degeneration (nAMD) | PHASE1 | Ocular Disorders |  |
| NCT06849609 | A Study to Evaluate the Tolerability, Safety and Efficacy of VGN-R13 in Patients with ALS | EARLY_PHASE1 | Neurological Disorders |  |
| NCT06308718 | Long-term Follow-up Study to Evaluate Safety and Efficacy of FBX-101 in Krabbe Patients |  | Neurological Disorders | rh10 |
| NCT06888661 | Clinical Trial to Assess the Safety and Efficacy of EXG001-307 in Patients With Spinal Muscular Atrophy | EARLY_PHASE1 | Neurological Disorders | 9 |
| NCT06942520 | Phase 2 Trial of RGX-314 in Adults With Center Involved - Diabetic Macular Edema (CI - DME) | PHASE2 | Ocular Disorders | 8 |
| NCT05876780 | A Gene Transfer Single Dose Study to Evaluate the Safety, Tolerability and Efficacy of SRP-9003 in Non-Ambulatory and Ambulatory Participants With Limb Girdle Muscular Dystrophy, Type 2E/R4 (Beta-Sarcoglycan [β-SG] Deficiency) | PHASE1 | Neurological Disorders | rh74 |
| NCT04273269 | A Safety and Efficacy Study of LYS-GM101 Gene Therapy in Patients With GM1 Gangliosidosis | PHASE1\|PHASE2 | Neurological Disorders | rh10 |
| NCT06971094 | Safety and Efficacy Evaluation of GC101 Gene Therapy Via Intrathecal (IT) Injectionin the Treatment of Patients With Type 2 Spinal Muscular Atrophy (SMA) - Phase III | PHASE3 | Neurological Disorders | 9 |
| NCT04903288 | A Study of SmartFlow Magnetic Resonance (MR) Compatible Ventricular Cannula for Administering Eladocagene Exuparvovec to Pediatric Participants | PHASE2 | Neurological Disorders | 2 |
| NCT04571970 | RGX-121 Gene Therapy in Children 5 Years of Age and Over With MPS II (Hunter Syndrome) | PHASE1\|PHASE2 | Metabolic Disorders | 9 |
| NCT07050160 | Long-term Follow-up Study of Gene Therapy for Arrhythmogenic Cardiomyopathy Due to a Plakophilin-2 Pathogenic Variant |  | Others | rh10 |
| NCT05820152 | Gene Therapy Clinical Trial for the Treatment of Leber's Hereditary Optic Neuropathy Associated With ND1 Mutations | PHASE1\|PHASE2 | Ocular Disorders | 2 |
| NCT06333249 | A Study Comparing Two Doses of AGTC-501 in Male Subjects With X-linked Retinitis Pigmentosa Caused by RPGR Mutations (SKYLINE) | PHASE2 | Ocular Disorders | 2 |
| NCT05293626 | Gene Therapy Clinical Trial for the Treatment of Leber's Hereditary Optic Neuropathy Associated With ND4 Mutations | PHASE1\|PHASE2 | Ocular Disorders | 2 |
| NCT06196840 | Safety and Efficacy of LX102 Gene Therapy in Patients With Neovascular Age-related Macular Degeneration (nAMD) (VENUS) | PHASE2 | Ocular Disorders | 2 |
| NCT05073133 | Safety and Efficacy of Intravenous OAV101 (AVXS-101) in Pediatric Patients With Spinal Muscular Atrophy (SMA) (OFELIA) | PHASE4 | Neurological Disorders | 9 |
| NCT05768386 | A Long-Term Follow-Up Study in Severe Hemophilia A Subjects Who Received BMN 270 in a Prior BioMarin Clinical Trial (270-401) |  | Blood Disorders | 5 |
| NCT06747273 | Study to Evaluate the Safety, Tolerability, and Efficacy of SRP-9004 Administered by Systemic Infusion in Limb Girdle Muscular Dystrophy Type 2D/R3 Participants in the United States | PHASE1 | Neurological Disorders | rh74 |
| NCT05164471 | Phase 1/2 Dose Confirmation Study of FLT180a in Hemophilia B | PHASE1\|PHASE2 | Blood Disorders | S3 (eng) |
| NCT05906251 | A Gene Transfer Study to Evaluate the Safety, Tolerability and Efficacy of SRP-6004 in Ambulatory Participants With Limb Girdle Muscular Dystrophy, Type 2B/R2 (LGMD2B/R2, Dysferlin [DYSF] Related) | PHASE1 | Neurological Disorders | rh74 |
| NCT04851873 | Safety and Efficacy of Intravenous OAV101 (AVXS-101) in Pediatric Patients With Spinal Muscular Atrophy (SMA) | PHASE3 | Neurological Disorders | 9 |
| NCT04597385 | Long-term Follow-Up for RGX-121 |  | Metabolic Disorders | 9 |
| NCT05629559 | 4D-310 in Adults With Fabry Disease and Cardiac Involvement | PHASE1\|PHASE2 | Metabolic Disorders | C102 (eng) |
| NCT05144386 | Study of EBT-101 in Aviremic HIV-1 Infected Adults on Stable ART | PHASE1 | Immune Disorders | 9 |
| NCT06952842 | Safety and Efficacy of ZVS203e in the Treatment of Retinitis Pigmentosa Caused by RHO Gene Mutation | PHASE1\|PHASE2 | Ocular Disorders | 8 |
| NCT06491927 | Long Term Follow-up for RGX-202 |  | Neurological Disorders | 8 |
| NCT05039866 | Long-Term Follow-up of Subjects Who Were Treated With ST-920 |  | Metabolic Disorders | 2/6 |
| NCT05514249 | Treatment of a Single Patient With CRD-TMH-001 | PHASE1 | Neurological Disorders | 9 |
| NCT06103487 | Long Term Follow-Up for RGX-111 |  | Metabolic Disorders | 9 |
| NCT04722107 | Safety Study of rAAV2/8-hCYP4V2 in Patients With Bietti's Crystalline Dystrophy (BCD) | EARLY_PHASE1 | Ocular Disorders | 2/8 (eng) |
| NCT06888492 | To Evaluate the Safety and Preliminary Efficacy of Intravitreal EXG202 Injection in Patients with Wet (neovascular) Age-related Macular Degeneration (wAMD) | EARLY_PHASE1 | Ocular Disorders |  |
| NCT06706427 | Safety and Efficacy Study of NGGT001 in Bietti Crystalline Corneoretinal Dystrophy Subjects | PHASE1\|PHASE2 | Ocular Disorders | 2 |
| NCT05752032 | A Long Term Follow-up Study of Subjects Who Received ICM-203 or Matching Placebo |  | Others | 1/2 |
| NCT06860672 | Clinical Trial of the Dual Vector Base Editor for the Treatment of the CHD3-R1025W Mutation | EARLY_PHASE1 | Neurological Disorders |  |
| NCT07054632 | Efficacy and Safety of LX101 for Inherited Retinal Dystrophy Associated With RPE65 Mutations | PHASE3 | Ocular Disorders | 2 |
| NCT06942572 | A Phase 1/2, First-in-Human Dose Escalation/Expansion Study to Evaluate the Safety, Tolerability and Preliminary Efficacy of a Subretinal Injection of SB-007 in Subjects With Stargardt Disease (STGD1) | PHASE1\|PHASE2 | Ocular Disorders | 8 |
| NCT06817343 | A Long-term Follow up Study of EXG102-031 in Patients With wAMD (Everest LTFU) | PHASE1 | Ocular Disorders | 8 |
| NCT06831825 | Study Assessing Left Ventricular Administration of a Genetic Medicine Directing Organ Regeneration in Heart Failure | PHASE1 | Others | 9 |
| NCT06196827 | Safety and Tolerability of LX101 for Inherited Retinal Dystrophy Associated With RPE65 Mutations | PHASE1\|PHASE2 | Ocular Disorders | 2 |
| NCT06241950 | A Gene Transfer Therapy Study to Evaluate the Safety and Efficacy of Delandistrogene Moxeparvovec (SRP-9001) Following Imlifidase Infusion in Participants With Duchenne Muscular Dystrophy (DMD) Determined to Have Pre-existing Antibodies to Recombinant Adeno-Associated Virus Serotype (rAAVrh74) | PHASE1 | Neurological Disorders | rh74 |
| NCT07064759 | Single Intravitreal Injection of 4D-150 in Patients With Macular Neovascularization Secondary to Age-Related Macular Degeneration | PHASE3 | Ocular Disorders | R100 (eng) |
| NCT05143307 | Long-Term Follow-Up Study of HIV-1 Infected Adults Who Received EBT-101 | PHASE1 | Immune Disorders | 9 |
| NCT05481827 | ORACLE: A Long-term Follow-up Study to Evaluate the Safety of GT005 in Participants With Geographic Atrophy Secondary to Age-related Macular Degeneration Treated in a Gyroscope-sponsored Antecedent Study | PHASE2 | Ocular Disorders | 2 |
| NCT05089656 | Efficacy and Safety of Intrathecal OAV101 (AVXS-101) in Pediatric Patients With Type 2 Spinal Muscular Atrophy (SMA) | PHASE3 | Neurological Disorders | 9 |
| NCT06483802 | A Study of ASP2016 in Adults Who Have Heart Disease Associated With Friedreich Ataxia | PHASE1 | Neurological Disorders | 8 |
| NCT05835895 | Safety and Pharmacodynamics of GNSC-001 Intra-articular Injection for Knee Osteoarthritis | PHASE1 | Others | 2.5 |
| NCT05429372 | Study of Fordadistrogene Movaparvovec in Early Stage Duchenne Muscular Dystrophy | PHASE2 | Neurological Disorders | 9 |
| NCT05243017 | Safety and Efficacy of AMT-130 in European Adults With Early Manifest Huntington's Disease | PHASE1\|PHASE2 | Neurological Disorders | 5 |
| NCT06864988 | 4D-150 in Patients With Macular Neovascularization Secondary to Age-Related Macular Degeneration | PHASE3 | Ocular Disorders | R100 (eng) |
| NCT07002398 | Safety and Preliminary Efficacy of VG801 in Patients With ABCA4 Mutation-associated Retinal Dystrophy (Stargardt Disease) | PHASE1\|PHASE2 | Ocular Disorders |  |
| NCT05778877 | A Study to Evaluate the Safety, Tolerability, and Pharmacodynamics of SEL-302 in Pediatric Subjects With MMA | PHASE1\|PHASE2 | Metabolic Disorders | 8 |
| NCT06844214 | A Study to Investigate the Safety, Tolerability, and Efficacy of SAR446268, an Adeno-associated Viral Vector-mediated Gene Therapy in Participants Aged 10 to 50 Years of Age With Non-congenital Myotonic Dystrophy Type 1 | PHASE1\|PHASE2 | Neurological Disorders | SAN011 |
| NCT07063251 | An Clinical Study Evaluating the Safety, Tolerability, and efficAcy of HG005 in StaRgardT Disease | EARLY_PHASE1 | Ocular Disorders |  |
| NCT05568719 | Safety and Effectiveness of Giroctocogene Fitelparvovec or Fidanacogene Elaparvovec in Patients With Hemophilia A or B Respectively | PHASE3 | Blood Disorders | 6 and Rh74 (eng) |
| NCT04411654 | Phase 1/2 Clinical Trial of PR001 in Infants With Type 2 Gaucher Disease (PROVIDE) | PHASE1\|PHASE2 | Metabolic Disorders | 9 |
| NCT05536973 | Safety and Efficacy of ADVM-022 in Treatment-Experienced Patients With Neovascular Age-related Macular Degeneration [LUNA] | PHASE2 | Ocular Disorders | 2 |
| NCT05386680 | Phase IIIb, Open-label, Multi-center Study to Evaluate Safety, Tolerability and Efficacy of OAV101 Administered Intrathecally to Participants With SMA Who Discontinued Treatment With Nusinersen or Risdiplam | PHASE3 | Neurological Disorders | 9 |
| NCT05607810 | Long-Term Follow-up Study of ADVM-022 in DME (INFINITY-EXT) |  | Ocular Disorders | 2 |
| NCT06856577 | Efficacy and Safety Study of Ixoberogene Soroparvovec (Ixo-vec) in Participants With Neovascular Age-Related Macular Degeneration | PHASE3 | Ocular Disorders | 7m8 (eng) |

*(eng), engineered

**Table S3.** Viral vector trials other than AAV.

| **NCT Number** | **Study Title** | **Delivery** | **Phases** | **Disease Category** |
| --- | --- | --- | --- | --- |
| **NCT04286815** | Gene Therapy for X Linked Severe Combined Immunodeficiency | Lenti |  | Immune Disorders |
| **NCT03603405** | HSV-tk and XRT and Chemotherapy for Newly Diagnosed GBM | Ad | PHASE1\|PHASE2 | Cancers |
| **NCT04601974** | Lentiviral Gene Therapy for Epilepsy | Lenti | PHASE1\|PHASE2 | Neurological Disorders |
| **NCT03596086** | HSV-tk + Valacyclovir + SBRT + Chemotherapy for Recurrent GBM | Ad | PHASE1\|PHASE2 | Cancers |
| **NCT03727555** | IT and IV Lentiviral Gene Therapy for X-ALD | Lenti |  | Neurological Disorders |
| **NCT03217617** | SCID-X1 Gene Therapy Via Intravenous Lentiviral (Ivlv-X1) Injection | Lenti | PHASE1\|PHASE2 | Immune Disorders |
| **NCT05686798** | Adenovirus Mediated Suicide Gene Therapy With Radiotherapy in Progressive Astrocytoma. | Ad | PHASE1 | Cancers |
| **NCT06731933** | Impact of COL7A1 Gene Therapy on SCC Recurrence in RDEB Skin | HSV | PHASE2 | Other |
| **NCT03645460** | Gene Therapy for ADA-SCID Using an Improved Lentiviral Vector (Ivlv-ADA) | Lenti |  | Immune Disorders |
| **NCT01913106** | HSV-tk + Valacyclovir Therapy in Combination With Brachytherapy for Recurrent Prostate Cancer | Ad | PHASE1\|PHASE2 | Cancers |
| **NCT06474442** | A Phase IIa Study of the Safety, Tolerability and Efficacy of BD111 in Herpes Simplex Virus Type I Stromal Keratitis | Lenti | PHASE2 | Other |
| **NCT06724900** | A Study Assessing KB304 for the Treatment of Wrinkles in Women | HSV | PHASE1\|PHASE2 | Other |
| **NCT05248789** | OH2 Oncolytic Viral Therapy in Advanced Bladder Cancer | HSV | PHASE2 | Cancers |
| **NCT05232136** | OH2 Oncolytic Viral Therapy in Non-Muscle-Invasive Bladder Cancer | HSV | PHASE1\|PHASE2 | Cancers |
| **NCT03866525** | OH2 Oncolytic Viral Therapy in Solid Tumors | HSV | PHASE1\|PHASE2 | Cancers |
| **NCT06504381** | DB107-RRV, DB107-FC, and Radiation Therapy with or Without Temozolomide (TMZ) for High Grade Glioma | Retro | PHASE1\|PHASE2 | Cancers |
| **NCT06757153** | Safety and Efficacy of NRG-103 Injection in the Treatment of Recurrent Glioblastoma Patients | Ad | EARLY_PHASE1 | Cancers |
| **NCT02705196** | LOAd703 Oncolytic Virus Therapy for Pancreatic Cancer | Ad | PHASE1\|PHASE2 | Cancers |
| **NCT06545955** | A Trial to Evaluate Intravesical Nadofaragene Firadenovec Alone or in Combination with Chemotherapy or Immunotherapy in Participants with High-grade BCG Unresponsive Non-muscle Invasive Bladder Cancer | Ad | PHASE2 | Cancers |
| **NCT06552598** | The Safety, Tolerability, and Efficacy of KD01 in Cervical Malignancies | Ad | PHASE1 | Cancers |
| **NCT05961111** | A Clinical Study on Oncolytic Virus Injection (R130 OV) for the Treatment of Advanced Solid Tumors | HSV | EARLY_PHASE1 | Cancers |
| **NCT05860374** | A Clinical Study on Oncolytic Virus Injection (R130) for the Treatment of Advanced Solid Tumors | HSV | EARLY_PHASE1 | Cancers |
| **NCT05801783** | A Clinical Study on Oncolytic Virus Injection (R130) for the Treatment of Relapsed/Refractory Ovarian Cancer | HSV | EARLY_PHASE1 | Cancers |
| **NCT05886075** | A Clinical Study on Oncolytic Virus Injection (R130) for the Treatment of Relapsed/Refractory Advanced Solid Tumors | HSV | EARLY_PHASE1 | Cancers |
| **NCT05830240** | A Clinical Study on Oncolytic Virus Injection (R130 OV) for the Treatment of Relapsed/Refractory Head and Neck Cancer | HSV | EARLY_PHASE1 | Cancers |
| **NCT06596681** | A Study of the Safety and Tolerability of GA in the Treatment of Patients with Refractory Neuropathic Pain | Lenti | EARLY_PHASE1 | Neurological Disorders |
| **NCT06510374** | Trial of Nadofaragene Firadenovec vs. Observation in Participants With Intermediate Risk Non-Muscle Invasive Bladder Cancer | Ad | PHASE3 | Cancers |
| **NCT06171282** | A Clinical Study on Oncolytic Virus Injection (R130) for the Treatment of Advanced Bone and Soft Tissue Tumors | HSV | EARLY_PHASE1 | Cancers |
| **NCT05812677** | A Clinical Study on Oncolytic Virus Injection (R130) for the Treatment of Relapsed/Refractory Cervical and Endometrial Cancer | HSV | EARLY_PHASE1 | Cancers |
| **NCT05851456** | A Clinical Study on Oncolytic Virus Injection (R130) for the Treatment of Relapsed/Refractory Bone and Soft Tissue Tumors | HSV | EARLY_PHASE1 | Cancers |
| **NCT06539338** | Safety of INT2104 in Participants Aged 18 Years and Older Who Have B-cell Cancer That Came Back After Prior Treatment | Lenti | PHASE1 | Cancers |
| **NCT04386967** | OH2 Injection in Solid Tumors | Pox | PHASE1\|PHASE2 | Cancers |
| **NCT04521764** | A Vaccine (MV-s-NAP) for the Treatment of Patients with Invasive Metastatic Breast Cancer | Pox | PHASE1 | Cancers |
| **NCT05684731** | Safety and Efficacy of KM1 in Subjects With Recurrent or Refractory Ovarian Cancer | Pox | PHASE1 | Cancers |
| **NCT03017820** | A Vaccine (VSV-hIFNŒ≤-NIS) with or Without Cyclophosphamide and Combinations of Ipilimumab, Nivolumab, and Cemiplimab in Treating Relapsed or Refractory Multiple Myeloma, Acute Myeloid Leukemia or Lymphoma | VSV | PHASE1 | Cancers |
| **NCT03767348** | Study of RP1 Monotherapy and RP1 in Combination With Nivolumab | HSV | PHASE2 | Other |
| **NCT05514990** | Bortezomib and Pembrolizumab With or Without Pelareorep for the Treatment of Relapsed or Refractory Multiple Myeloma, AMBUSH Trial | Reovirus | PHASE1\|PHASE2 | Cancers |
| **NCT06508463** | Intravenous Vesicular Stomatitis Virus in Patients With Peripheral T-cell Lymphoma | VSV | PHASE1 | Cancers |
| **NCT05914376** | Safety of Recombinant Human IL-21-expressing Oncolytic Vaccinia Virus Injection (hV01) in Advanced Tumors | Pox | PHASE1 | Cancers |
| **NCT05393440** | First-in-human (FIH) Phase I Trial of BS-006 in Cervical Cancer | HSV | PHASE1 | Cancers |
| **NCT04911166** | Phase I Trial of Atezolizumab and Interleukin-12 Gene Therapy in Metastatic Non-Small Cell Lung Cancer With Progression on First-Line Immunotherapy With or Without Chemotherapy | Ad | PHASE1 | Cancers |
| **NCT04095689** | Docetaxel Chemotherapy and Pembrolizumab Plus Interleukin-12 Gene Therapy in Triple Negative Breast Cancer | Ad | PHASE2 | Cancers |
| **NCT03725670** | Direct Lentiviral Injection Gene Therapy for MLD | Lenti |  | Neurological Disorders |
| **NCT05223725** | Gene Therapy for Post-Operative Atrial Fibrillation | Ad | PHASE1 | Others |
| **NCT06834438** | Gene Therapy for Neurofibromatosis Type 2 (NF2) with ST002 | Lenti |  | Neurological Disorders |
| **NCT04378244** | CORONA: A Study Using DeltaRex-G Gene Therapy for Symptomatic COVID-19 | Retro | PHASE1\|PHASE2 | Others |
| **NCT05099094** | VEGFA-targeting Gene Therapy to Treat Retinal and Choroidal Neovascularization Diseases | Lenti | EARLY_PHASE1 | Ocular Disorders |
| **NCT04739046** | An Exploratory Trial to Evaluate Efficacy and Safety for Combination Treatment of Adenovirus Double Suicide Gene Therapy | Ad | PHASE2 | Cancers |
| **NCT05743270** | Study of RP3 in Combination With Nivolumab and Other Therapy in Patients With Locoregionally Advanced or Recurrent SCCHN | HSV | PHASE2 | Cancers |
| **NCT06356701** | Tumor Nutritional Therapy in the First-line Treatment of Stage IV NSCLC | Lenti | PHASE1 | Others |
| **NCT04917887** | Long-Term Follow-up Protocol | HSV |  | Others (genetic skin disorder) |
| **NCT06884865** | A Study to Assess Safety and Tolerability of PCRX-201 in Subjects With Painful Osteoarthritis of the Knee | Ad | PHASE2 | Others |
| **NCT05733611** | RP2/RP3 in Combination With Atezolizumab and Bevacizumab for the Treatment of Patients With CRC | HSV | PHASE2 | Cancers |
| **NCT06914479** | Virus-Based Gene Therapy (AdV-HSV1-TK and AdV-Flt3L) in Combination With Valacyclovir for the Treatment of Pediatric and Young Adult Patients With Resectable, Recurrent Primary Malignant Brain Tumors | Ad | PHASE1 | Cancers |
| **NCT07006077** | Recombinant Human IL-21-expressing Oncolytic Vaccinia Virus Injection (hV01) in Advanced Pancreatic Cancer | Pox | PHASE2 | Cancers |
| **NCT04741061** | Study to Evaluate Efficacy, Immunogenicity and Safety of the Sputnik-Light | Ad | PHASE3 | Immune Disorders |
| **NCT06962852** | A Long-term Study to Monitor the Health Status of People With Cystic Fibrosis Who Took Part in a Previous Study With BI 3720931 (Lenticlair™-ON) | Lenti | PHASE1\|PHASE2 | Metabolic Disorders |
| **NCT06515002** | A Study to Test How Well BI 3720931 is Tolerated and Whether it Improves Lung Function in People With Cystic Fibrosis (Lenticlair™ 1) | Lenti | PHASE1\|PHASE2 | Metabolic Disorders |
| **NCT06887348** | A Study to Assess the Long-term Safety Outcomes in Patients Previously Treated With RP1, RP2, or RP3 | HSV |  | Cancers |
